# Transfer and Fitness of IS*Aba52*-Mediated *tet*(X3) Transposon in *Acinetobacter* spp.

**DOI:** 10.3390/microorganisms13122656

**Published:** 2025-11-22

**Authors:** Chong Chen, Jing Liu, Jie Gao, Yubing Hua, Taotao Wu, Jinlin Huang

**Affiliations:** 1 Joint International Research Laboratory of Agriculture and Agri-Product Safety, Ministry of Education of China, Institutes of Agricultural Science and Technology Development, Yangzhou University, Yangzhou 225009, China; 2 Jiangsu Key Laboratory of Zoonosis, Jiangsu Co-Innovation Center for Prevention and Control of Important Animal Infectious Diseases and Zoonoses, Yangzhou University, Yangzhou 225009, China; 3 Key Laboratory of Prevention and Control of Biological Hazard Factors (Animal Origin) for Agrifood Safety and Quality, Ministry of Agriculture of China, Yangzhou University, Yangzhou 225009, China; 4 College of Veterinary Medicine, Yangzhou University, Yangzhou 225009, China

**Keywords:** *Acinetobacter* spp., *tet*(X3), IS*Aba52*, multidrug resistance, bacterial fitness

## Abstract

The global spread of tigecycline resistance conferred by *tet*(X3) poses a serious threat to clinical treatment of multidrug-resistant (MDR) *Acinetobacter* infections. Despite *tet*(X3) being detected in diverse *Acinetobacter* species, its transposition mechanism and fitness in these pathogens remain poorly characterized. Here, we reported the first plasmid-borne IS*Aba52*-mediated transposable element harboring *tet*(X3) in *Acinetobacter amyesii* YH16040. Conjugation experiments demonstrated the transferability of *tet*(X3) into the chromosome of *Acinetobacter baylyi* ADP1 at an efficiency of (7.1 ± 2.5) × 10^−8^. High-throughput sequencing revealed six tandem copies of IS*Aba52*-flanked *tet*(X3), *floR*, and *sul2* forming a 231.6 Kb complex transposon in the obtained transconjugant *A. baylyi* YH16040C. Phenotypic assays showed that YH16040C exhibited elevated resistance to tigecycline, chlortetracycline, florfenicol, and trimethoprim-sulfamethoxazole by 64- to 256-fold. Notably, YH16040C exhibited a growth advantage, reduced competition ability, and non-significant difference in biofilm formation compared to ADP1 in antibiotic-free backgrounds. Under moderate antibiotic treatment of tigecycline, chlortetracycline, florfenicol, and trimethoprim-sulfamethoxazole, the competition ability of YH16040C against ADP1 was significantly higher than that without antibiotics. All of these highlight the importance of IS*Aba52*-mediated transposition in disseminating *tet*(X3) between *Acinetobacter* species and elucidate the fitness changes employed by MDR strains under antibiotic selection pressures. Our study advocates the urgent need for surveillance of IS*Aba52*-associated resistance elements in human, animal, and environmental settings.

## 1. Introduction

Tigecycline is the third-generation tetracycline antibiotic, acting as a last-resort approach to treat clinical MDR bacterial infections [1,2]. Since its first approval by the U.S. Food and Drug Administration (FDA) in 2015, tigecycline-resistant Gram-negative and Gram-positive bacteria have been inevitably detected worldwide [3,4,5]. The tigecycline resistance is usually conferred by bacterial efflux pumps and ribosomal protection proteins, including *adeABC*, *tmexCD-toprJ*, *tet*(A), *tet*(L), and *tet*(M) [6,7,8,9,10]. By contrast, the flavin-dependent monooxygenase Tet(X) can degrade all tetracycline antibiotics by hydroxylation, representing a unique enzymatic inactivation mechanism [11]. Recently, the rapid spread of the mobile tigecycline resistance gene *tet*(X3) has raised the concern that tigecycline may be clinically ineffective [12,13]. Thus far, the *tet*(X3) gene has been detected in *Acinetobacter* sp. isolates from various ecological niches in humans, food-producing animals, vegetables, soils, and water, especially in China [12,14,15]. Nevertheless, the horizontal transmission mechanism of *tet*(X3) in *Acinetobacter* spp. deserves further study.

The genus *Acinetobacter* is a heterogeneous group of Gram-negative bacteria, comprising 90 validly published species and more than 70 unnamed ones [16,17]. They are ubiquitous in nature but can also cause serious infections in hospital settings, including pneumonia, bloodstream infections, and wound infections [18,19,20,21]. In 2024, carbapenem-resistant *Acinetobacter baumannii* (CRAB) was classified as the critical group by the World Health Organization (WHO) Bacterial Priority Pathogens List [22]. Worrisomely, the *tet*(X3)-mediated tigecycline resistance and *bla*_NDM-1_-mediated carbapenem-resistance were simultaneously detected in *A*. *baumannii*, *Acinetobacter indicus*, *Acinetobacter schindleri*, *Acinetobacter lwoffii*, *Acinetobacter johnsonii*, *Acinetobacter portensis*, and *Acinetobacter junii* [12,14,23,24]. Although plasmid- and chromosome-borne *tet*(X3) genes have been reported, genetic environments indicated they were closely related to the insertion sequence IS*CR2*, possibly promoting the dissemination of multiple antibiotic resistance genes (ARGs) such as *tet*(X3), *sul2*, and *floR* [14,25]. On the other hand, the acquisition of ARGs usually leads to the fitness change of bacterial growth, competition, and biofilms, and antibiotics are important factors for adaptability, which remain poorly understood for *tet*(X3) [26,27,28].

As one of the strictly aerobic, oxidase-negative, and catalase-positive *Acinetobacter* species, *A*. *amyesii* has been reported in animal, soil, and water samples in the Czech Republic, Germany, Turkey, and Indonesia since its public report in 2022 [29,30]. In this study, we first reported an IS*Aba52*-mediated transposon of *tet*(X3) between *A. amyesii* and *A*. *baylyi* in China by conjugation and high-throughput sequencing, and then explored the bacterial fitness by antimicrobial susceptibility, growth, competition, and biofilm experiments under the selection pressure of tigecycline, chlortetracycline, florfenicol, and trimethoprim-sulfamethoxazole.

## 2. Materials and Methods

### 2.1. Bacterial Strains and Ethical Statement

All procedures involving animal-derived isolates were approved by the Institutional Animal Care and Use Committee of Yangzhou University under the protocol number 202403072, following the relevant biosafety and ethical requirements. The *tet*(X3)-positive *A. amyesii* strain YH16040 was isolated from pig manure in 2016 in Jiangxi, China, derived from our previous multiregional study of *tet*(X)-mediated tigecycline-resistant *Acinetobacter* spp. [14]. *A. baylyi* ADP1, *Escherichia coli* C600, *E. coli* 25922, and *Salmonella typhi* ATCC 14028 are standard strains preserved in our laboratory.

### 2.2. Conjugation Experiments

The transferability of *tet*(X3)-mediated tigecycline resistance in *A. amyesii* YH16040 was determined by filter mating with rifampicin-resistant recipient strains *A. baylyi* ADP1, *E. coli* C600, and *S. typhi* ATCC 14028. After static incubation with the 0.22 μm pore-size filter membrane on Luria–Bertani (LB) agar plates at a donor/recipient ratio of 1/3 at 37 °C for 16 h, the mixture was resuspended in 1 mL LB broth. The putative transconjugants of them were all spread and selected on LB agar plates containing tigecycline (2 µg/mL) and rifampin (120 µg/mL), with tigecycline inhibiting the recipient strains and rifampicin inhibiting the donor strain in the bacterial mixture. Then they were screened for the target gene *tet*(X3) by PCR and confirmed by PCR-fingerprints for *A. baylyi* or enterobacterial repetitive intergenic consensus PCR (ERIC-PCR) for *E. coli* and *S. typhi* using genomic DNA as the templates [14]. Meanwhile, the recipient cells were spread and selected on LB agar plates containing rifampin (120 µg/mL). Transfer efficiency was calculated with mean ± standard deviation (SD) based on visual colony counts of the transconjugant and recipient cells [31].

### 2.3. Whole Genome Sequencing (WGS) and Bioinformatics Analyses

The bacterial genome was extracted using the TIANamp Bacteria DNA Kit (Tiangen, Beijing, China). To obtain the complete circular structures, WGS was performed by Illumina HiSeq and Oxford Nanopore sequencing (BENAGEN, Wuhan, China), followed by assembly with Unicycler version 0.5.0 and correction with Pilon version 1.24 [32,33]. Genome quality evaluation was conducted by QUAST version 5.2.0 and CheckM version 1.1.6, with the parameters of contigs (<300), N50 (>50 kb), completeness (>95%), contamination (<2%), and heterogeneity (<50%) [34,35]. Gene prediction and annotation were performed by Rapid Annotation using Subsystem Technology (RAST) version 2.0 [36]. Insertion sequences were analyzed by the online ISfinder platform [37]. The visual representation of the *tet*(X3)-related transposition process was generated with Easyfig version 2.2.5 [38]. Additionally, the 705 bp open reading frame (ORF) of IS*Aba52* of *Acinetobacter* spp. (taxid: 469) and *E. coli* (taxid: 562) deposited in the National Center for Biotechnology Information (NCBI) WGS and core_nt databases were collected by blastn, with a threshold of 100% nucleotide sequence similarity and coverage. Compared with the whole-genome sequences of *Acinetobacter* and *E. coli* type strains (https://lpsn.dsmz.de/, accessed on 10 November 2025), an average nucleotide identity (ANI) analysis was conducted using FastANI version 1.3 to define precise bacterial species by >95% [39].

### 2.4. Antimicrobial Susceptibility Testing

According to the U.S. Clinical and Laboratory Standards Institute (CLSI) guideline, minimum inhibitory concentrations (MICs) of chlortetracycline (0.125–256 μg/mL), tigecycline (0.015625–32 μg/mL), florfenicol (0.125–256 μg/mL), trimethoprim-sulfamethoxazole (1.25–320 μg/mL), CuSO_4_ (7.8125–2000 μg/mL), and CdCl_2_ (3.125–800 μg/mL) were determined by two-fold broth dilution in 200 μL Mueller–Hinton (MH) broth in 96-well plates at 37 °C for 24 h, respectively [40]. Particularly, the breakpoints of chlortetracycline, florfenicol, and trimethoprim-sulfamethoxazole were interpreted by CLSI, and that of tigecycline was interpreted by the FDA criterion for *Enterobacteriaceae* bacteria, which should be re-adjusted for MIC interpretation if the tigecycline resistance breakpoint for *Acinetobacter* spp. was established [14,40]. No clinical breakpoints were available for heavy metals CuSO_4_ and CdCl_2_. *E. coli* 25922 was used as the quality control strain.

### 2.5. Growth Curves

Suspensions of *A. baylyi* ADP1 and its transconjugant in the logarithmic growth phase were adjusted to a uniform concentration (OD_600_ = 0.1) for the determination of bacterial growth curves, respectively. After dilution with sterilized LB broth at a volume/volume ratio of 1/100, they were incubated with three different concentrations of tigecycline (0.015625, 0.125, and 1 μg/mL), chlortetracycline (0.125, 1, and 8 μg/mL), florfenicol (0.25, 1, and 4 μg/mL), or trimethoprim-sulfamethoxazole (0.625, 2.5, and 10 μg/mL) at 37 °C and 200 rpm for 16 h, and the antibiotic-untreated bacterial strains were used as blank controls. Particularly, the concentrations of four antibiotics were selected based on the results of MICs. OD_600_ was measured by Multifunctional Microplate Reader (BioTek Instruments, Inc., Winooski, VT, USA) at 0 h, 1 h, 2 h, 4 h, 6 h, 8 h, 12 h, and 16 h.

### 2.6. Competition Experiments

Suspensions of *A. baylyi* ADP1 and its transconjugant in the logarithmic growth phase (OD_600_ = 0.1) were mixed at a volume/volume ratio of 1/1, and then incubated with sub-inhibitory concentrations of tigecycline (0.015625 μg/mL), chlortetracycline (0.125 μg/mL), florfenicol (0.25 μg/mL), or trimethoprim-sulfamethoxazole (0.625 μg/mL) in LB broth at 37 °C and 200 rpm for 48 h. The antibiotic-untreated bacterial strains were used as blank controls. After a series of 10-fold dilutions, the transconjugant and *A. baylyi* ADP1 from the mixture were spread and selected by LB agar plates containing tigecycline (2 μg/mL) or not and confirmed by PCR amplification of the target gene *tet*(X3) using genomic DNA as the templates at 0 h, 24 h and 48 h, respectively [41]. As previously reported, the relative fitness (RF) was calculated with mean ± SD using the formula [RF = (log_10_S1_dt_ − log_10_S1_d0_)/(log_10_S2_dt_ − log_10_S2_d0_)] [42,43]. S1 and S2 represented visual colony-forming unit (CFU) densities of the transconjugant and *A. baylyi* ADP1, respectively. dt and d0 were the tested days. RF > 1 indicated that the transconjugant had a selective advantage over *A. baylyi* ADP1, whereas RF < 1 was defined as the bacterial fitness cost.

### 2.7. Crystal Violet Staining

After static incubation with three different concentrations of tigecycline (0.015625, 0.125, and 1 μg/mL), chlortetracycline (0.125, 1, and 8 μg/mL), florfenicol (0.25, 1, and 4 μg/mL), or trimethoprim-sulfamethoxazole (0.625, 2.5, and 10 μg/mL) in LB broth at 37 °C for 24 h, the bacterial suspension of *A. baylyi* ADP1 or its transconjugant was discarded. The antibiotic-untreated bacterial strains were used as blank controls. Next, the precipitation was washed three times with phosphate buffer saline (PBS), fixed in methanol for 15 min, stained with 0.1% crystal violet for 15 min, and then washed three times with PBS. Following the treatment of 95% ethanol for 15 min, OD_570_ was measured for the bacterial biofilm by Multifunctional Microplate Reader.

### 2.8. Statistical Analyses

All the bacterial conjugation, susceptibility, growth, competition, and biofilm experiments were performed in three biological replicates and repeated on at least two independent occasions. Statistical analyses were calculated by an unpaired and two-tailed *t*-test using GraphPad Prism version 8.3.0. Significant difference was defined as follows: *, *p* < 0.05; **, *p* < 0.01; ***, *p* < 0.001; ****, *p* < 0.0001; Not significant (NS), *p* ≥ 0.05.

## 3. Results

### 3.1. Transferability of tet(X3)-Mediated Tigecycline Resistance

In our previous national investigation, a *tet*(X3)-positive tigecycline-resistant *Acinetobacter* sp. YH16040 was isolated from a pig in 2016 in Jiangxi, China [14]. Here, it was re-designated as *A. amyesii* by ANI analysis with its type strain, namely *A. amyesii* ANC 5579 (GenBank accession number: GCA_023499985.1). MICs of *A. amyesii* YH16040 indicated that it was resistant to tigecycline (8 µg/mL), chlortetracycline (64 µg/mL), florfenicol (32 µg/mL), and trimethoprim-sulfamethoxazole (160 µg/mL) (Table 1). Successfully, the *tet*(X3) gene was transferred into the recipient *A. baylyi* ADP1 by conjugation at a frequency of (7.1 ± 2.5) × 10^−8^, suggesting its transferability. MIC results showed the obtained transconjugant YH16040C exhibited chlortetracycline resistance (64 μg/mL) and tigecycline resistance (8 μg/mL), together with the resistance to florfenicol (64 μg/mL) and trimethoprim-sulfamethoxazole (160 μg/mL), which increased by 64- to 256-fold compared with those of *A. baylyi* ADP1 (Table 1). However, there is no difference in MICs of heavy metals CuSO_4_ (500 μg/mL) or CdCl_2_ (12.5 μg/mL) between *A. baylyi* ADP1 and YH16040C (Table 1). On the other hand, the conjugation experiments with the recipient *E. coli* C600 and *S. typhi* ATCC 14028 failed despite multiple attempts, confirming that the genus *Acinetobacter* is a more appropriate bacterial host of *tet*(X3) than *Enterobacteriaceae*.

### 3.2. Transposition Mechanism of tet(X3) Across Acinetobacter Species

**Figure 1:**
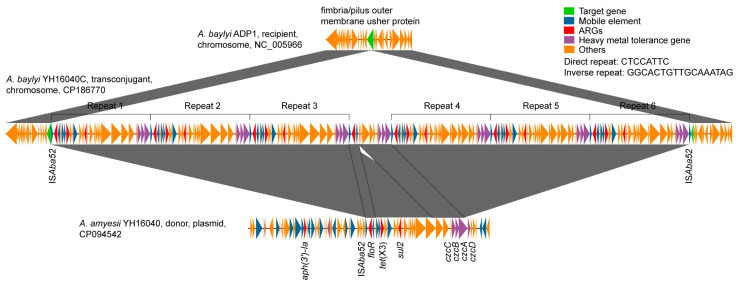
IS*Aba52*-mediated transposition of *tet*(X3) in *Acinetobacter* species. The target gene, mobile element, ARGs, heavy metal tolerance gene, and other genes are represented by green, blue, red, purple, and orange colors, respectively. Regions of >98% nucleotide identity are marked by shading.

*A. amyesii* YH16040 carried a single combination of tigecycline resistance gene *tet*(X3), florfenicol resistance gene *floR*, trimethoprim-sulfamethoxazole resistance gene *sul2*, aminoglycoside resistance gene *aph(3’)-la*, and heavy metal tolerance genes (*czcA*, *czcB*, *czcC*, and *czcD*) on an 87.4 Kb GR31 circular plasmid (CP094542) [44]. WGS analyses of its transconjugant *A. baylyi* YH16040C were also conducted in this study. Genome quality met the requirements for the next analyses, such as the average depth (441×), contig (*n* = 1), N50 (3,833,693 kb), completeness (100%), contamination (0%), and heterogeneity (0%). Annotation results of the genetic environment revealed six IS*Aba52*-mediated tandem repeats of *tet*(X3), *floR*, *sul2*, *czcA*, *czcB*, and *czcC* on the circular chromosome of YH16040C (CP186770), sharing >99.9% nucleotide identity and >99.9% nucleotide coverage. Presumably, a 35.9 Kb transposable unit from the donor *A. amyesii* YH16040 was inserted into the chromosome-derived fimbria/pilus outer membrane usher gene of *A. baylyi* ADP1 (NC_005966) six times; In addition, an IS*Aba52*-mediated 15.4 Kb transposon of *floR*, *czcA*, *czcB*, and *czcC* was also found, resulting in the final 231.6 Kb complex transposon (Figure 1). GC content of the complex transposon (45.4%) was higher than that of the chromosome of *A. baylyi* ADP1 (40.4%). However, there was no significant additive effect on MICs of antibiotics or heavy metals between *A. baylyi* YH16040C and *A. amyesii* YH16040 (Table 1).

As described in the ISfinder database, IS*Aba52* belongs to the IS*6* family and is 820 bp in length, containing an ORF from 64 bp to 768 bp and encoding a 234 aa transposase protein that shares 98% aa similarity to IS*1006*. Despite sporadic reports, results of the blastn query on 45,274 *Acinetobacter* genomes in the NCBI WGS database and 118,620,752 nucleotide sequences in the NCBI core_nt database further identified 781 non-duplicate bacterial strains carrying IS*Aba52*, of which three isolates carried two copies each. For the bacterial species by ANI analyses, IS*Aba52* was widely distributed in 20 different *Acinetobacter* species (Table S1). The majority was *A. baumannii* (*n* = 688), followed by *Acinetobacter nosocomialis* (*n* = 22), *A. amyesii* (*n* = 15), *A. indicus* (*n* = 11), *Acinetobacter pittii* (*n* = 7), *Acinetobacter towneri* (*n* = 5), Taxon 76 (*n* = 5), *Acinetobacter pseudolwoffii* (*n* = 5), *Acinetobacter ursingii* (*n* = 4), *A. johnsonii* (*n* = 4), *Acinetobacter bereziniae* (*n* = 2), *A. schindleri* (*n* = 2), *Acinetobacter variabilis* (*n* = 2), *Acinetobacter yuyunsongii* (*n* = 2), Taxon 58 (*n* = 2), *A. lwoffii* (*n* = 1), *Acinetobacter faecalis* (*n* = 1), *Candidatus Acinetobacter avistercoris* (*n* = 1), Taxon 83 (*n* = 1), and novel Taxon 96 (*n* = 1). However, none was detected in 337,799 *E. coli* genomes in the NCBI WGS database and 118,620,752 nucleotide sequences in the NCBI core_nt database.

### 3.3. Fitness Effect of ISAba52-Mediated tet(X3) Transposon

#### 3.3.1. Bacterial Growth

**Figure 2:**
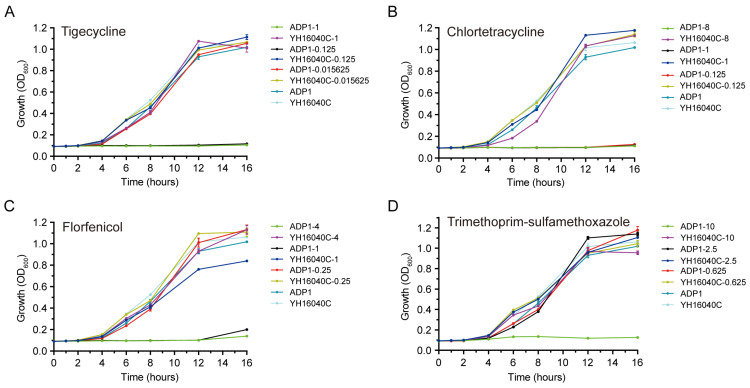
Bacterial growth curve. The growth curve under the selection pressure of tigecycline (**A**), chlortetracycline (**B**), florfenicol (**C**), and trimethoprim-sulfamethoxazole (**D**) is present, respectively. The *A. baylyi* strains YH16040C (transconjugant) and ADP1 (recipient) without antibiotics are used as the blank controls.

To explore the growth effect of IS*Aba52*-mediated *tet*(X3) transposon, growth curves of *A. baylyi* ADP1 and its transconjugant YH16040C were determined under different concentrations of tigecycline, chlortetracycline, florfenicol, and trimethoprim-sulfamethoxazole, respectively (Figure 2). After four hours of co-culture, most of the tested strains rapidly proliferated except ADP1 inhibited by relatively high concentrations. In a blank control, YH16040C exhibited a significantly higher growth advantage than that of ADP1 at 16 h (*p* = 0.0268). Following incubation with a low dose of tigecycline (0.015625 μg/mL, *p* = 0.3929), florfenicol (0.25 μg/mL, *p* = 0.7018), or trimethoprim-sulfamethoxazole (0.625 μg/mL, *p* = 0.0498; 2.5 μg/mL, *p* = 0.1722), the growth rate of ADP1 reached that of YH16040C at 16 h. With the concentration increased, the growth advantage of YH16040C was all significantly higher than that of ADP1 at 16 h (*p* < 0.0001). These data showed that bacterial strains obtaining the IS*Aba52*-mediated *tet*(X3) transposon have a growth advantage, especially under moderate antibiotic treatment.

#### 3.3.2. Bacterial Competition

**Figure 3:**
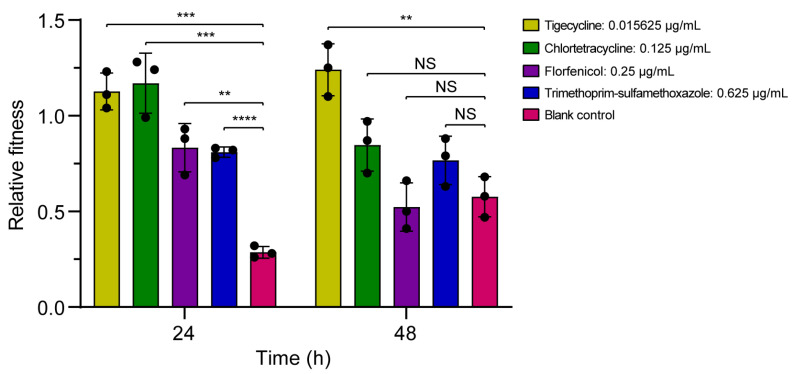
Relative fitness under the selection of tigecycline, chlortetracycline, florfenicol, and trimethoprim-sulfamethoxazole. *A. baylyi* ADP1 and its transconjugant YH16040C without antibiotics are used as the blank controls. **, *p* < 0.01; ***, *p* < 0.001; ****, *p* < 0.0001; NS, not significant, *p* ≥ 0.05.

Furthermore, the competitive differentiation between *A. baylyi* ADP1 and YH16040C under the treatment of four antibiotics was analyzed (Figure 3). In the absence of antibiotics, YH16040C has a fitness cost compared to *A. baylyi* ADP1 (RF < 1). With the extension of incubation time, the fitness cost at 48 h (RF = 0.58 ± 0.09) was alleviated compared to that at 24 h (RF = 0.29 ± 0.02). Under the incubation of tigecycline (0.015625 μg/mL), YH16040C had a higher fitness advantage at 48 h (RF = 1.24 ± 0.11) than that at 24 h (RF = 1.13 ± 0.08). For chlortetracycline (0.125 μg/mL), it exhibited a similar advantage at 24 h (RF = 1.17 ± 0.13) but lost it at 48 h (RF = 0.85 ± 0.11). However, with the addition of florfenicol (0.25 μg/mL) or trimethoprim-sulfamethoxazole (0.625 μg/mL), YH16040C had a fitness cost at both 24 h and 48 h, with RF values ranging from 0.52 ± 0.1 to 0.83 ± 0.1.

At 24 h, all of tigecycline (*p* = 0.0001), chlortetracycline (*p* = 0.0007), florfenicol (*p* = 0.0019), and trimethoprim-sulfamethoxazole (*p* < 0.0001) were significantly beneficial for the competition ability of *A. baylyi* YH16040C carrying IS*Aba52*-mediated *tet*(X3) transposon than that without antibiotics. At 48 h, only tigecycline (*p* = 0.0026) was significantly beneficial, while chlortetracycline (*p* = 0.0533), florfenicol (*p* = 0.6044), and trimethoprim-sulfamethoxazole (*p* = 0.1161) were not. The results indicated that antibiotics, even at low concentrations, may promote the competition ability of bacterial strains with transposons of *tet*(X3), especially tigecycline.

#### 3.3.3. Bacterial Biofilm

**Figure 4:**
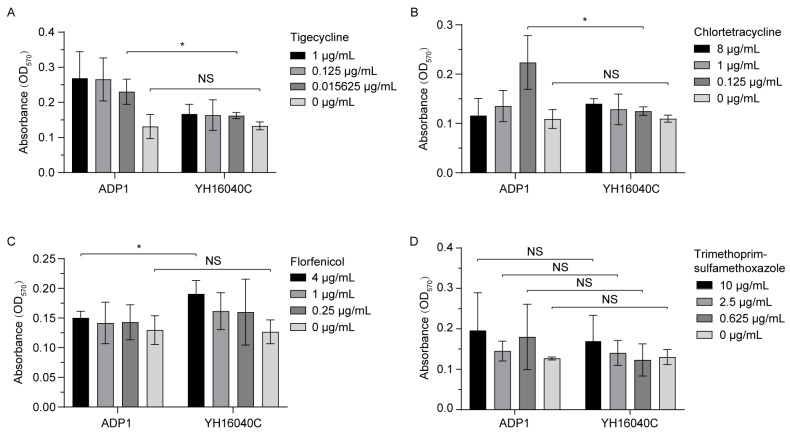
Bacterial biofilms. The biofilm under the selection pressure of tigecycline (**A**), chlortetracycline (**B**), florfenicol (**C**), and trimethoprim-sulfamethoxazole (**D**) is present, respectively. *A. baylyi* ADP1 and its transconjugant YH16040C, grown without antibiotics, serve as the blank controls. *, *p* < 0.05; NS, not significant, *p* ≥ 0.05.

In addition, the bacterial biofilm was successfully determined by the crystal violet staining method under different antibiotic selection pressure, resulting in complex phenotypic changes (Figure 4). Initially, there was no significant difference in biofilm formation between *A. baylyi* ADP1 and YH16040C in the absence of antibiotics (*p* ≥ 0.05). Under the induction of antibiotics, the biofilm of YH16040C was significantly lower than that of ADP1 at an appropriate concentration of tigecycline (0.015625 μg/mL, *p* = 0.0336) or chlortetracycline (0.125 μg/mL, *p* = 0.0364), while YH16040C was significantly higher than ADP1 at 4 μg/mL of florfenicol (*p* = 0.0494). With the addition of trimethoprim-sulfamethoxazole, the biofilm formation of YH16040C exhibited no significant change (*p* ≥ 0.05) compared to ADP1 under equivalent concentration conditions.

## 4. Discussion

To the best of our knowledge, *tet*(X3) is a unique tigecycline resistance gene in *Acinetobacter* sp. bacteria from human, animal, and environmental samples [15,45]. Previous studies showed that it was usually associated with IS*CR2*, which is an IS*91*-like element that lacks the inverted repeat sequences of most IS elements, and was located in a mobile transposon designed as IS*CR2*-*tpnF*-*tet*(X3)-hp-hp-IS*CR2* [14,46]. In this study, we first reported an IS*Aba52*-mediated transposition of *tet*(X3) between *A. amyesii* and *A. baylyi*. In silico analysis also revealed IS*Aba52* was widely distributed but restricted in *Acinetobacter* species, including *A. baumannii*, *A. nosocomialis*, *A. amyesii*, *A. indicus*, *A. pittii*, *A. towneri*, *A. pseudolwoffii*, *A. ursingii*, *A. johnsonii*, *A. bereziniae*, *A. schindleri*, *A. variabilis*, *A. yuyunsongii*, *A. lwoffii*, *A. faecalis*, *Candidatus A. avistercoris*, and four undefined species (namely Taxon 58, Taxon 76, Taxon 83, and Taxon 96). All these data indicate there is a risk of horizontal transmission of IS*Aba52*-mediated *tet*(X3) gene in *Acinetobacter* species, which was underestimated previously, and tigecycline resistance monitoring and control are urgently needed following the international consensus of One Health.

Since the first report in 2019 in China, multiple copies of *tet*(X3) tandem structures have been detected in *A. baumannii*, *A. indicus*, *A. pseudolwoffii*, *A. variabilis*, and *A. schindleri* [12,14,44,47]. It is noted that *tet*(X3) and *tet*(X6) were also simultaneously detected in multiple *Acinetobacter* species, such as *A. indicus*, *A. pseudolwoffii*, *A. schindleri*, *A. towneri*, *A. amyesii*, *A. lwoffii*, and *A. variabilis* [14,15,23,48]. Here, we reported six IS*Aba52*-mediated tandem repeats of ARGs *tet*(X3), *floR*, and *sul2* on the chromosome of transconjugant *A. baylyi* YH16040C. However, there was no significant additive effect on MICs of tigecycline, chlortetracycline, florfenicol, or trimethoprim-sulfamethoxazole between *A. baylyi* YH16040C and its donor *A. amyesii* YH16040, which was consistent with our previous study on cloning of *tet*(X3) and *tet*(X6) [14]. Many studies indicated the tandem amplification was primarily achieved through complex gene rearrangements mediated by insertion sequence (IS), leading to an increase in the copy number of resistance genes and thereby forming heterogeneous resistant subpopulations among cells [49,50,51]. The molecular mechanism underlying the *tet*(X3), *floR*, and *sul2* redundancy may be explained by the high propensity of *Acinetobacter* species to acquire *tet*(X3), *floR*, and *sul2* genes by IS*Aba52*, and remains to be further studied.

Acquired antibiotic resistance will provide adequate protection in the antibiotic environment, which may also cause bacterial metabolic burden and fitness cost if antibiotic use is inhibited [52,53]. After incubation in the blank medium, the relative fitness of *A. baumannii* obtaining a *tet*(X3)-positive plasmid or *E. coli* and *K. pneumoniae* obtaining a *tet*(X4)-positive plasmid was approximately 0.7–0.8 [12]. With the addition of different concentrations of L-arabinose, the fitness cost of *E. coli* or *Salmonella Enteritidis* carrying an arabinose-inducible plasmid pBAD-*tet*(X6) was significantly higher than that of *Proteus mirabilis* [54]. In this study, the capture of IS*Aba52*-mediated *tet*(X3) in *A. baylyi* YH16040C led to a growth benefit, reduced competition ability, and non-significant difference in biofilm formation compared to *A. baylyi* ADP1 in the absence of antibiotics. The reduced competition ability despite growth benefits can be attributed to several factors. First, trade-offs between the growth rate and other fitness traits may occur, where the rapid growth compromises the competition ability [55]. Second, metabolic costs associated with growth-enhancing adaptations may divert resources from competition functions [56]. Third, environmental factors like nutrient limitation or spatial structure can decouple growth rates from competitive outcomes [57]. Apparently, the balance between benefits and fitness costs ultimately determines bacterial survival and evolutionary trajectories across diverse ecosystems.

A low dose of clinical antibiotics is often detected in humans, animals, and natural environments, representing an important risk factor affecting bacterial survival [58,59,60,61]. As previously reported, the specific resistance mechanism of tetracyclines was selected by each antibiotic generation [62]. The adaptability of *A. baumannii* under sub-MIC pressure of tigecycline was closely associated with a resistance-nodulation-division (RND) efflux pump AdeABC [8]. At present, we explored the effect of tetracyclines (chlortetracycline, tigecycline), phenicols (florfenicol), and sulphonamides (trimethoprim-sulfamethoxazole) on bacterial fitness. Under moderate antibiotic treatment, the competition ability of *A. baylyi* YH16040C against *A. baylyi* ADP1 was significantly higher than that without antibiotics, suggesting antibiotics promote the spread of *tet*(X)-positive *Acinetobacter* sp. strains. Although tigecycline is only approved to be used in human infections, tetracycline, florfenicol, and trimethoprim-sulfamethoxazole are largely utilized for animal production and human health [63,64]. Inevitably, *Acinetobacter* spp. carrying *tet*(X) genes exhibited multidrug resistance to the commonly used quinolone (e.g., ciprofloxacin) and carbapenem (e.g., meropenem) antibiotics, which contributed to their further spread [14,24,44]. In addition, heavy metals are also important environmental contaminants for co-selection of antibiotic resistance [65,66]. But the tolerance genes found in this study did not mediate the phenotype, which is worth further exploration. Therefore, limiting the use of antibiotics is critical in controlling the emergence and spread of multiple antimicrobial resistance in *Acinetobacter* sp. pathogens.

## 5. Conclusions

This study elucidated the transposition mechanism and fitness effect of mobile *tet*(X3) in *Acinetobacter* species. We identified the first IS*Aba52*-mediated transposon harboring *tet*(X3), *floR*, and *sul2* in *A. amyesii*, which exhibited efficient horizontal transferability into *A. baylyi* via conjugation. The transconjugant displayed elevated resistance to tigecycline, chlortetracycline, florfenicol, and trimethoprim-sulfamethoxazole, driven by tandem IS*Aba52* repeats forming a complex transposable unit. Antibiotic-dependent advantages of *tet*(X3)-harboring bacteria outcompeting *tet*(X3)-negative parental strains was revealed under moderate antibiotic treatment. These findings highlight the role of IS*Aba52*-mediated transposition in disseminating *tet*(X3) and the potential fitness changes of *Acinetobacter* spp. due to antibiotic selection. Surveillance of such elements is critical to mitigate the spread of multidrug resistance in human, animal, and environmental settings.

## Figures and Tables

**Table 1:** MICs of the tetracyclines, phenicols, sulfonamides, and heavy metals (μg/mL).

Strains	Tigecycline	Chlortetracycline	Florfenicol	Trimethoprim-Sulfamethoxazole ^a^	CuSO_4_	CdCl_2_
*A. amyesii* YH16040	8	64	32	160	500	12.5
*A. baylyi* YH16040C	8	64	64	160	500	12.5
*A. baylyi* ADP1	0.03125	0.25	1	2.5	500	12.5

^a^ Trimethoprim and sulfamethoxazole are mixed at a ratio of 1/19 before two-fold broth dilution.

## Data Availability

The original contributions presented in this study are included in the article/Supplementary Materials. Further inquiries can be directed to the corresponding author.

## Supplementary Materials

The following supporting information can be downloaded at: https://www.mdpi.com/article/10.3390/microorganisms13122656/s1, Table S1: ANI-based *Acinetobacter* species carrying IS*Aba52*.

## Abbreviations

The following abbreviations are used in this manuscript:

MDR	Multidrug-resistant
FDA	Food and Drug Administration
CRAB	Carbapenem-resistant *Acinetobacter baumannii*
WHO	World Health Organization
ARGs	Antibiotic resistance genes
LB	Luria–Bertani
ERIC-PCR	Enterobacterial repetitive intergenic consensus PCR
SD	Standard deviation
WGS	Whole genome sequencing
RAST	Rapid annotation using subsystem technology
ORF	Open reading frame
NCBI	National Center for Biotechnology Information
ANI	Average nucleotide identity
CLSI	Clinical and Laboratory Standards Institute
MICs	Minimum inhibitory concentrations
MH	Mueller–Hinton
RF	Relative fitness
CFU	Colony-forming unit
PBS	Phosphate buffer saline
NS	Not significant
IS	Insertion sequence
RND	Resistance-nodulation-division
