# Supplementary material for: Transfer and Fitness of ISAba52-Mediated tet(X3) Transposon in Acinetobacter spp
Source: Microorganisms. 2025 Nov 22;13(12):2656. doi: 10.3390/microorganisms13122656 (PMC12734723; doi:10.3390/microorganisms13122656)
Supplement: Supplementary file 1 [file microorganisms-13-02656-s001.zip › microorganisms-3947467-supplementary.pdf]

Order	Species (ANI)	Description	Coverage	Identity	Accession
1	*Acinetobacter amyesii*	Acinetobacter sp. YH1901130 NODE_42_length_24005_cov_132.738002	100%	100%	VPEV01000044.1
2	*Acinetobacter amyesii*	Acinetobacter sp. YH16056 NODE_163_length_1124_cov_4313.51	100%	100%	VPER01000161.1
3	*Acinetobacter amyesii*	Acinetobacter sp. YH16055 NODE_123_length_2366_cov_13735.752121	100%	100%	VPEQ01000126.1
4	*Acinetobacter amyesii*	Acinetobacter sp. YH16040 NODE_56_length_10607_cov_222.624237	100%	100%	VPEI01000057.1
5	*Acinetobacter amyesii*	Acinetobacter sp. YH16037 NODE_157_length_1391_cov_2.238924	100%	100%	VPEF01000159.1
6	*Acinetobacter amyesii*	Acinetobacter sp. YH12086 NODE_36_length_6322_cov_277.821146	100%	100%	VPAF01000037.1
7	*Acinetobacter amyesii*	Acinetobacter sp. YH12080 NODE_35_length_41692_cov_175.856057	100%	100%	VPAA01000036.1
8	*Acinetobacter amyesii*	Acinetobacter sp. YH12079 NODE_26_length_10531_cov_143.096886	100%	100%	VOZZ01000026.1
9	*Acinetobacter amyesii*	Acinetobacter sp. YH12076 NODE_28_length_10531_cov_111.997789	100%	100%	VOZY01000028.1
10	*Acinetobacter amyesii*	Acinetobacter sp. YH12054 NODE_36_length_5998_cov_61.498041	100%	100%	VOZJ01000037.1
11	*Acinetobacter amyesii*	Acinetobacter sp. YH12049 NODE_41_length_21281_cov_30.288172	100%	100%	VOZH01000041.1
12	*Acinetobacter amyesii*	Acinetobacter sp. YH12036 NODE_60_length_13143_cov_198.230101	100%	100%	VOYZ01000062.1
13	*Acinetobacter amyesii*	Acinetobacter sp. YH12023 NODE_22_length_8862_cov_322.374471	100%	100%	VOYU01000024.1
14	*Acinetobacter amyesii*	Acinetobacter sp. YH12068_T plasmid pYH12068-5, complete sequence	100%	100%	CP094560.1
15	*Acinetobacter amyesii*	Acinetobacter sp. SH20PTE14 chromosome, complete genome	100%	100%	CP090067.1
16	*Acinetobacter baumannii*	Acinetobacter baumannii strain WM97a NODE_52_length_9678_cov_17.055561	100%	100%	JACYYA010000052.1
17	*Acinetobacter baumannii*	Acinetobacter baumannii strain VNBM-AB253-2 SAMD00556425-rid21364723.denovo.065	100%	100%	DAXVCP010000060.1
18	*Acinetobacter baumannii*	Acinetobacter baumannii strain VNBM-AB253-1 SAMD00556424-rid21362853.denovo.028	100%	100%	DAXUYU010000028.1
19	*Acinetobacter baumannii*	Acinetobacter baumannii strain VNBM-AB222-2 SAMD00556380-rid21364633.denovo.087	100%	100%	DAXVPI010000087.1
20	*Acinetobacter baumannii*	Acinetobacter baumannii strain VNBM-AB189-3 SAMD00556330-rid21364513.denovo.002	100%	100%	DAXVDA010000002.1
21	*Acinetobacter baumannii*	Acinetobacter baumannii strain VNBM-AB189-2 SAMD00556329-rid21361333.denovo.112	100%	100%	DAXUZL010000112.1
22	*Acinetobacter baumannii*	Acinetobacter baumannii strain VNBM-AB189-1 SAMD00556328-rid21364503.denovo.010	100%	100%	DAXVBB010000010.1
23	*Acinetobacter baumannii*	Acinetobacter baumannii strain SK016 NODE_29_length_5160_cov_81.700964	100%	100%	JAIGUF010000029.1
24	*Acinetobacter baumannii*	Acinetobacter baumannii strain SIMBA027 2	100%	100%	JBJLVH010000002.1
25	*Acinetobacter baumannii*	Acinetobacter baumannii strain RIVM_C059494 SAMN49342092-rid26759373.denovo.15	100%	100%	DBJEXX010000014.1
26	*Acinetobacter baumannii*	Acinetobacter baumannii strain RIVM_C048201 SAMN49342055-rid26759393.denovo.66	100%	100%	DBJEXW010000059.1
27	*Acinetobacter baumannii*	Acinetobacter baumannii strain RIVM_C048196 SAMN49342054-rid26757523.denovo.13	100%	100%	DBJEVM010000013.1
28	*Acinetobacter baumannii*	Acinetobacter baumannii strain RIVM_C046913 SAMN49342043-rid26759113.denovo.65	100%	100%	DBJEYS010000058.1
29	*Acinetobacter baumannii*	Acinetobacter baumannii strain RIVM_C015536 SAMN49342035-rid26757223.denovo.095	100%	100%	DBJEYR010000088.1
30	*Acinetobacter baumannii*	Acinetobacter baumannii strain PSU043 NODE_30_length_5160_cov_68.153846	100%	100%	JAIGYH010000030.1
31	*Acinetobacter baumannii*	Acinetobacter baumannii strain MyJU123 SAMD00178090-rid8882893.denovo.30	100%	100%	DADAUX010000030.1
32	*Acinetobacter baumannii*	Acinetobacter baumannii strain MRSN2024 SAMN04491037-rid12666443.denovo.13	100%	100%	AAYNNH020000012.1
33	*Acinetobacter baumannii*	Acinetobacter baumannii strain MRSN2021 SAMN04480662-rid12665573.denovo.37	100%	100%	AAYNRB020000030.1
34	*Acinetobacter baumannii*	Acinetobacter baumannii strain Microbial isolate ESKAPEE pathogen SAMN28613889-rid14588723.denovo.43	100%	100%	DAHKXI010000043.1
35	*Acinetobacter baumannii*	Acinetobacter baumannii strain J15 J15.fa_65_length_18219_cov_21.081837	100%	100%	VKGL01000082.1
36	*Acinetobacter baumannii*	Acinetobacter baumannii strain gs43 gs43_56	100%	100%	JAUGXU010000056.1
37	*Acinetobacter baumannii*	Acinetobacter baumannii strain G1728 17161_contig_143	100%	100%	JASGXF010000143.1
38	*Acinetobacter baumannii*	Acinetobacter baumannii strain E314 NODE_55_length_4550_cov_22.257744	100%	100%	JAEHMK010000055.1
39	*Acinetobacter baumannii*	Acinetobacter baumannii strain E0321 EO321_c0056	100%	100%	JALJDV010000056.1
40	*Acinetobacter baumannii*	Acinetobacter baumannii strain BZU_Abaum_15 NODE_101_length_31652_cov_156.651367	100%	100%	JALBWN010000089.1
41	*Acinetobacter baumannii*	Acinetobacter baumannii strain BAuABod-3 NODE_60_length_23964_cov_36.2082	100%	100%	NIWN01000060.1
42	*Acinetobacter baumannii*	Acinetobacter baumannii strain ARC7019 ARC7019_contig_152	100%	100%	JAUENN010000152.1
43	*Acinetobacter baumannii*	Acinetobacter baumannii strain ARC6801 ARC6801_contig_53	100%	100%	JAUEOP010000053.1
44	*Acinetobacter baumannii*	Acinetobacter baumannii strain AR9254 contig00055	100%	100%	JBKFLG010000055.1
45	*Acinetobacter baumannii*	Acinetobacter baumannii strain Acinetobacter baumanii 255 SAMEA2376395-rid7072693.denovo.55	100%	100%	DADBCX010000045.1
46	*Acinetobacter baumannii*	Acinetobacter baumannii strain Aci00859 Aci00859-merged_contig_59	100%	100%	VAGE01000059.1
47	*Acinetobacter baumannii*	Acinetobacter baumannii strain Ab121 genome assembly, contig: NODE_109_length_11823_cov_16.385605	100%	100%	UCPB02000060.1
48	*Acinetobacter baumannii*	Acinetobacter baumannii strain A9108 Scaffold32_1	100%	100%	JAHTNM010000035.1
49	*Acinetobacter baumannii*	Acinetobacter baumannii strain A522 3	100%	100%	JACEIJ010000003.1
50	*Acinetobacter baumannii*	Acinetobacter baumannii strain A423 2	100%	100%	JACEIG010000002.1
51	*Acinetobacter baumannii*	Acinetobacter baumannii strain A217 3	100%	100%	JACEIH010000003.1
52	*Acinetobacter baumannii*	Acinetobacter baumannii strain A134 SAMN13028174-rid9741963.guided.055	100%	100%	DADAQB010000099.1
52	*Acinetobacter baumannii*	Acinetobacter baumannii strain A134 SAMN13028174-rid9741963.denovo.056	100%	100%	DADAQB010000051.1
53	*Acinetobacter baumannii*	Acinetobacter baumannii strain 81-58664-AB SAMD00555760-rid21362043.denovo.152	100%	100%	DAXVCH010000145.1
54	*Acinetobacter baumannii*	Acinetobacter baumannii strain 48_n NODE_62_length_20823_cov_26.265497	100%	100%	JAWXYJ010000062.1
55	*Acinetobacter baumannii*	Acinetobacter baumannii strain 4300STDY7045874 genome assembly, contig: ERS1930352SCcontig000039	100%	100%	UFOI01000039.1
56	*Acinetobacter baumannii*	Acinetobacter baumannii strain 4300STDY7045872 genome assembly, contig: ERS1930350SCcontig000029	100%	100%	UFOH01000029.1
57	*Acinetobacter baumannii*	Acinetobacter baumannii strain 4300STDY7045852 genome assembly, contig: ERS1930325SCcontig000039	100%	100%	UFNO01000039.1
58	*Acinetobacter baumannii*	Acinetobacter baumannii strain 4300STDY7045846 genome assembly, contig: ERS1930319SCcontig000086	100%	100%	UISY01000086.1
59	*Acinetobacter baumannii*	Acinetobacter baumannii strain 4300STDY7045845 genome assembly, contig: ERS1930318SCcontig000036	100%	100%	UFNM01000036.1
60	*Acinetobacter baumannii*	Acinetobacter baumannii strain 2025HL-01512 isolate IDR2400079336-01-00 SAMN46427897-rid24953853.denovo.09	100%	100%	ABXFXP010000009.1
61	*Acinetobacter baumannii*	Acinetobacter baumannii strain 2025HL-00541 isolate IDR2500048257-01-00 SAMN49975366-rid26903943.denovo.09	100%	100%	ABZRVB010000009.1
62	*Acinetobacter baumannii*	Acinetobacter baumannii strain 2025HL-00496 isolate IDR2500042094-01-00 SAMN49654326-rid26759953.denovo.09	100%	100%	ABZLLQ010000009.1
63	*Acinetobacter baumannii*	Acinetobacter baumannii strain 2025HL-00495 isolate IDR2500041978-01-00 SAMN49654325-rid26758103.denovo.09	100%	100%	ABZLLR010000009.1
64	*Acinetobacter baumannii*	Acinetobacter baumannii strain 2025HL-00493 isolate IDR2500041665-01-02 SAMN49654323-rid26759853.denovo.10	100%	100%	ABZLMA010000010.1
65	*Acinetobacter baumannii*	Acinetobacter baumannii strain 2025HL-00490 isolate IDR2500040982-01-02 SAMN49654320-rid26757993.denovo.10	100%	100%	ABZLLZ010000010.1
66	*Acinetobacter baumannii*	Acinetobacter baumannii strain 2025HL-00488 isolate IDR2500040980-01-02 SAMN49654318-rid26759873.denovo.08	100%	100%	ABZLLV010000008.1
67	*Acinetobacter baumannii*	Acinetobacter baumannii strain 2025HL-00486 isolate IDR2500040978-01-02 SAMN49654316-rid26758063.denovo.09	100%	100%	ABZLLS010000009.1
68	*Acinetobacter baumannii*	Acinetobacter baumannii strain 2025HL-00480 isolate IDR2500040533-01-00 SAMN49639478-rid26753353.denovo.10	100%	100%	ABZLKI010000010.1
69	*Acinetobacter baumannii*	Acinetobacter baumannii strain 2025HL-00473 isolate IDR2500038588-01-00 SAMN49639471-rid26753303.denovo.09	100%	100%	ABZLLG010000009.1
70	*Acinetobacter baumannii*	Acinetobacter baumannii strain 2025HL-00470 isolate IDR2500037565-01-02 SAMN49639468-rid26753373.denovo.05	100%	100%	ABZLKK010000005.1
71	*Acinetobacter baumannii*	Acinetobacter baumannii strain 2025HL-00469 isolate IDR2500040320-01-00 SAMN49102619-rid26704003.denovo.12	100%	100%	ABZJFH010000012.1
72	*Acinetobacter baumannii*	Acinetobacter baumannii strain 2025HL-00449 isolate IDR2500037558-01-02 SAMN49005314-rid26678433.denovo.09	100%	100%	ABZHSG010000009.1
73	*Acinetobacter baumannii*	Acinetobacter baumannii strain 2025HL-00448 isolate IDR2500037552-01-02 SAMN49005313-rid26677903.denovo.09	100%	100%	ABZHIJ010000009.1
74	*Acinetobacter baumannii*	Acinetobacter baumannii strain 2025HL-00446 isolate IDR2500037504-01-00 SAMN49005311-rid26678443.denovo.04	100%	100%	ABZHQE010000004.1
75	*Acinetobacter baumannii*	Acinetobacter baumannii strain 2025HL-00445 isolate IDR2500037250-01-00 SAMN49005310-rid26677933.denovo.10	100%	100%	ABZHII010000010.1
76	*Acinetobacter baumannii*	Acinetobacter baumannii strain 2025HL-00444 isolate IDR2500037229-01-00 SAMN49005309-rid26678453.denovo.09	100%	100%	ABZHLM010000009.1
77	*Acinetobacter baumannii*	Acinetobacter baumannii strain 2025HL-00439 isolate IDR2500037213-01-00 SAMN48791041-rid26540753.denovo.13	100%	100%	ABZDLG010000013.1
78	*Acinetobacter baumannii*	Acinetobacter baumannii strain 2025HL-00412 isolate IDR2500034616-01-00 SAMN48681485-rid26491213.denovo.59	100%	100%	ABZBSR010000053.1
79	*Acinetobacter baumannii*	Acinetobacter baumannii strain 2025HL-00409 isolate IDR2500033412-01-00 SAMN48537708-rid26441933.denovo.11	100%	100%	ABYZMF010000011.1
80	*Acinetobacter baumannii*	Acinetobacter baumannii strain 2025HL-00408 isolate IDR2500033394-01-01 SAMN48537707-rid26441943.denovo.09	100%	100%	ABYZMJ010000009.1
81	*Acinetobacter baumannii*	Acinetobacter baumannii strain 2025HL-00400 isolate IDR2500032621-01-00 SAMN48525078-rid26432223.denovo.10	100%	100%	ABYZFU010000010.1
82	*Acinetobacter baumannii*	Acinetobacter baumannii strain 2025HL-00397 isolate IDR2500031960-01-00 SAMN48525075-rid26432823.denovo.33	100%	100%	ABYZFN010000029.1
83	*Acinetobacter baumannii*	Acinetobacter baumannii strain 2025HL-00396 isolate IDR2500031942-01-00 SAMN48525074-rid26432043.denovo.10	100%	100%	ABYZFT010000010.1
84	*Acinetobacter baumannii*	Acinetobacter baumannii strain 2025HL-00390 isolate IDR2500030874-01-00 SAMN48525068-rid26431993.denovo.07	100%	100%	ABZAFE010000007.1
85	*Acinetobacter baumannii*	Acinetobacter baumannii strain 2025HL-00389 isolate IDR2500030641-01-02 SAMN48525067-rid26432183.denovo.10	100%	100%	ABYZFX010000010.1
86	*Acinetobacter baumannii*	Acinetobacter baumannii strain 2025HL-00384 isolate IDR2500030133-01-00 SAMN48525062-rid26432213.denovo.10	100%	100%	ABYZFV010000010.1
87	*Acinetobacter baumannii*	Acinetobacter baumannii strain 2025HL-00383 isolate IDR2500030132-01-02 SAMN48525061-rid26432023.denovo.09	100%	100%	ABZAFA010000009.1
88	*Acinetobacter baumannii*	Acinetobacter baumannii strain 2025HL-00367 isolate IDR2500030105-01-00 SAMN48206988-rid26221813.denovo.11	100%	100%	ABYUWL010000011.1
89	*Acinetobacter baumannii*	Acinetobacter baumannii strain 2025HL-00360 isolate IDR2500028317-01-00 SAMN48100329-rid26110393.denovo.09	100%	100%	ABYSZN010000009.1
90	*Acinetobacter baumannii*	Acinetobacter baumannii strain 2025HL-00358 isolate IDR2500027870-01-00 SAMN48100327-rid26110413.denovo.12	100%	100%	ABYSZL010000012.1
91	*Acinetobacter baumannii*	Acinetobacter baumannii strain 2025HL-00284 isolate IDR2500026380-01-02 SAMN48033515-rid26067553.denovo.10	100%	100%	ABYROL010000010.1
92	*Acinetobacter baumannii*	Acinetobacter baumannii strain 2025HL-00269 isolate IDR2500024597-01-02 SAMN47937978-rid26013663.denovo.10	100%	100%	ABYQNG010000010.1
93	*Acinetobacter baumannii*	Acinetobacter baumannii strain 2025HL-00268 isolate IDR2500024507-01-02 SAMN47937977-rid26021953.denovo.10	100%	100%	ABYQPC010000010.1
94	*Acinetobacter baumannii*	Acinetobacter baumannii strain 2025HL-00262 isolate IDR2500024106-01-00 SAMN47937971-rid26018533.denovo.26	100%	100%	ABYQPB010000026.1
95	*Acinetobacter baumannii*	Acinetobacter baumannii strain 2025HL-00261 isolate IDR2500023473-01-02 SAMN47937970-rid26013693.denovo.09	100%	100%	ABYQND010000009.1
96	*Acinetobacter baumannii*	Acinetobacter baumannii strain 2025HL-00259 isolate IDR2500023029-01-01 SAMN47937968-rid26018493.denovo.06	100%	100%	ABYTMQ010000006.1
97	*Acinetobacter baumannii*	Acinetobacter baumannii strain 2025HL-00256 isolate IDR2500022248-01-02 SAMN47937965-rid26013623.denovo.10	100%	100%	ABYQNH010000010.1
98	*Acinetobacter baumannii*	Acinetobacter baumannii strain 2025HL-00255 isolate IDR2500022243-01-02 SAMN47937964-rid26021913.denovo.10	100%	100%	ABYQPH010000010.1
99	*Acinetobacter baumannii*	Acinetobacter baumannii strain 2025HL-00234 isolate IDR2500022240-01-02 SAMN47784884-rid25867503.denovo.09	100%	100%	ABYNYS010000009.1
100	*Acinetobacter baumannii*	Acinetobacter baumannii strain 2025HL-00217 isolate IDR2500019835-01-02 SAMN47572699-rid25798423.denovo.11	100%	100%	ABYKBU010000011.1
101	*Acinetobacter baumannii*	Acinetobacter baumannii strain 2025HL-00216 isolate IDR2500019631-01-01 SAMN47572698-rid25798433.denovo.09	100%	100%	ABYKBT010000009.1
102	*Acinetobacter baumannii*	Acinetobacter baumannii strain 2025HL-00204 isolate IDR2500019600-01-00 SAMN47442457-rid25717083.denovo.09	100%	100%	ABYJNU010000009.1
103	*Acinetobacter baumannii*	Acinetobacter baumannii strain 2025HL-00188 isolate IDR2500016118-01-00 SAMN47384031-rid25650993.denovo.12	100%	100%	ABYHIP010000012.1
104	*Acinetobacter baumannii*	Acinetobacter baumannii strain 2025HL-00180 isolate IDR2500016400-01-00 SAMN47383462-rid25649853.denovo.10	100%	100%	ABYHKB010000010.1
105	*Acinetobacter baumannii*	Acinetobacter baumannii strain 2025HL-00143 isolate IDR2500011000-01-00 SAMN47002955-rid25448443.denovo.10	100%	100%	ABYBPV010000010.1
106	*Acinetobacter baumannii*	Acinetobacter baumannii strain 2025HL-00107 isolate IDR2500007640-01-00 SAMN46999271-rid25446403.denovo.10	100%	100%	ABYBNP010000010.1
107	*Acinetobacter baumannii*	Acinetobacter baumannii strain 2025HL-00097 isolate IDR2500005837-01-02 SAMN46999261-rid25445163.denovo.09	100%	100%	ABYBHL010000009.1
108	*Acinetobacter baumannii*	Acinetobacter baumannii strain 2025HL-00096 isolate IDR2500005461-01-02 SAMN46999260-rid25445173.denovo.09	100%	100%	ABYBHJ010000009.1
109	*Acinetobacter baumannii*	Acinetobacter baumannii strain 2025HL-00046 isolate IDR2500006286-01-00 SAMN46755705-rid25282233.denovo.09	100%	100%	ABXRCK010000009.1
110	*Acinetobacter baumannii*	Acinetobacter baumannii strain 2025HL-00044 isolate IDR2500006247-01-00 SAMN46755703-rid25280563.denovo.15	100%	100%	ABXRCJ010000015.1
111	*Acinetobacter baumannii*	Acinetobacter baumannii strain 2025HL-00042 isolate IDR2500005149-01-00 SAMN46755701-rid25282263.denovo.10	100%	100%	ABXRCH010000010.1
112	*Acinetobacter baumannii*	Acinetobacter baumannii strain 2025HL-00041 isolate IDR2500005148-01-00 SAMN46755700-rid25282273.denovo.10	100%	100%	ABXRCF010000010.1
113	*Acinetobacter baumannii*	Acinetobacter baumannii strain 2025HL-00035 isolate IDR2500003818-01-00 SAMN46755694-rid25280543.denovo.09	100%	100%	ABXRCR010000009.1
114	*Acinetobacter baumannii*	Acinetobacter baumannii strain 2025HL-00032 isolate IDR2500000840-01-00 SAMN46755691-rid25282193.denovo.10	100%	100%	ABXRCP010000010.1
115	*Acinetobacter baumannii*	Acinetobacter baumannii strain 2025HL-00029 isolate IDR2400079327-01-00 SAMN46755688-rid25282223.denovo.34	100%	100%	ABXRCN010000031.1

116	*Acinetobacter baumannii*	Acinetobacter baumannii strain 2025HL-00004 isolate IDR2400078806-01-00 SAMN46429621-rid24956003.denovo.09	100%	100%	ABXFZP010000009.1
117	*Acinetobacter baumannii*	Acinetobacter baumannii strain 2025GO-0201 SAMN48207110-rid26222053.denovo.12	100%	100%	ABYUWB010000012.1
118	*Acinetobacter baumannii*	Acinetobacter baumannii strain 2025GO-0200 SAMN48207109-rid26222063.denovo.58	100%	100%	ABYUWA010000053.1
119	*Acinetobacter baumannii*	Acinetobacter baumannii strain 2025GO-0199 SAMN48207108-rid26222073.denovo.09	100%	100%	ABYUVZ010000009.1
120	*Acinetobacter baumannii*	Acinetobacter baumannii strain 2025GO-0170 SAMN48441780-rid26393373.denovo.26	100%	100%	ABYYIX010000026.1
121	*Acinetobacter baumannii*	Acinetobacter baumannii strain 2025GO-0144 SAMN47814511-rid25955593.denovo.16	100%	100%	ABYOTT010000016.1
122	*Acinetobacter baumannii*	Acinetobacter baumannii strain 2025GO-0103 SAMN48121359-rid26125423.denovo.03	100%	100%	ABYTFG010000003.1
123	*Acinetobacter baumannii*	Acinetobacter baumannii strain 2025GO-00293 SAMN49879516-rid26856683.denovo.42	100%	100%	ABZRPU010000035.1
124	*Acinetobacter baumannii*	Acinetobacter baumannii strain 2025EP-00218 SAMN49006010-rid26679003.denovo.12	100%	100%	ABZHTY010000012.1
125	*Acinetobacter baumannii*	Acinetobacter baumannii strain 2025EP-00098 SAMN47639649-rid25841433.denovo.27	100%	100%	ABYMFE010000025.1
126	*Acinetobacter baumannii*	Acinetobacter baumannii strain 2025EP-00062 SAMN47227034-rid25558473.denovo.14	100%	100%	ABYKIC010000014.1
127	*Acinetobacter baumannii*	Acinetobacter baumannii strain 2025EP-00007 SAMN46898456-rid25348323.denovo.16	100%	100%	ABYAPE010000016.1
128	*Acinetobacter baumannii*	Acinetobacter baumannii strain 2024HL-01454 isolate IDR2400072508-01-00 SAMN45960649-rid24608923.denovo.10	100%	100%	ABWXPZ010000010.1
129	*Acinetobacter baumannii*	Acinetobacter baumannii strain 2024HL-01452 isolate IDR2400072249-01-00 SAMN45960647-rid24608943.denovo.12	100%	100%	ABWXPY010000012.1
130	*Acinetobacter baumannii*	Acinetobacter baumannii strain 2024HL-01440 isolate IDR2400071301-01-00 SAMN45960635-rid24609043.denovo.10	100%	100%	ABWXPS010000010.1
131	*Acinetobacter baumannii*	Acinetobacter baumannii strain 2024HL-01337 1	100%	100%	ABWRYX020000001.1
132	*Acinetobacter baumannii*	Acinetobacter baumannii strain 2024HL-01334 contig00001	100%	100%	ABWRVD020000001.1
133	*Acinetobacter baumannii*	Acinetobacter baumannii strain 2024HL-01319 isolate IDR2400069715-01-00 SAMN45458558-rid24452523.denovo.02	100%	100%	ABWQWB010000002.1
134	*Acinetobacter baumannii*	Acinetobacter baumannii strain 2024HL-01230 isolate IDR2400062242-01-00 SAMN44652780-rid23949323.denovo.09	100%	100%	ABWIQL010000009.1
135	*Acinetobacter baumannii*	Acinetobacter baumannii strain 2024HL-01224 isolate IDR2400061545-01-02 SAMN44652774-rid23937873.denovo.08	100%	100%	ABWIIN010000008.1
136	*Acinetobacter baumannii*	Acinetobacter baumannii strain 2024HL-01223 isolate IDR2400061215-01-02 SAMN44652773-rid23937113.denovo.09	100%	100%	ABWIIM010000009.1
137	*Acinetobacter baumannii*	Acinetobacter baumannii strain 2024HL-01216 isolate IDR2400061911-01-00 SAMN44603251-rid23921993.denovo.009	100%	100%	ABWFRH010000009.1
138	*Acinetobacter baumannii*	Acinetobacter baumannii strain 2024HL-01208 isolate IDR2400061373-01-00 SAMN44603243-rid23922573.denovo.09	100%	100%	ABWFTI010000009.1
139	*Acinetobacter baumannii*	Acinetobacter baumannii strain 2024HL-01206 isolate IDR2400061268-01-00 SAMN44603241-rid23922583.denovo.10	100%	100%	ABWGAX010000010.1
140	*Acinetobacter baumannii*	Acinetobacter baumannii strain 2024HL-01205 isolate IDR2400061267-01-00 SAMN44603240-rid23922593.denovo.09	100%	100%	ABWFPI010000009.1
141	*Acinetobacter baumannii*	Acinetobacter baumannii strain 2024HL-01200 isolate IDR2400060586-01-03 SAMN44603235-rid23922413.denovo.12	100%	100%	ABWFRJ010000012.1
142	*Acinetobacter baumannii*	Acinetobacter baumannii strain 2024HL-01185 isolate IDR2400059707-01-00 SAMN44457064-rid23698033.denovo.09	100%	100%	ABWARE010000009.1
143	*Acinetobacter baumannii*	Acinetobacter baumannii strain 2024HL-01156 isolate IDR2400059731-01-00 SAMN44347234-rid23647783.denovo.09	100%	100%	ABVYTT010000009.1
144	*Acinetobacter baumannii*	Acinetobacter baumannii strain 2024HL-01151 isolate IDR2400057972-01-02 SAMN44347229-rid23647693.denovo.11	100%	100%	ABVYTY010000011.1
145	*Acinetobacter baumannii*	Acinetobacter baumannii strain 2024HL-01140 isolate IDR2400058302-01-00 SAMN44333245-rid23635043.denovo.10	100%	100%	ABVXOP010000010.1
146	*Acinetobacter baumannii*	Acinetobacter baumannii strain 2024HL-01139 isolate IDR2400058301-01-00 SAMN44333244-rid23635053.denovo.11	100%	100%	ABVYTH010000011.1
147	*Acinetobacter baumannii*	Acinetobacter baumannii strain 2024HL-01123 isolate IDR2400056092-01-01 SAMN44333228-rid23634983.denovo.11	100%	100%	ABVXOS010000011.1
148	*Acinetobacter baumannii*	Acinetobacter baumannii strain 2024HL-01115 isolate IDR2400056806-01-00 SAMN44099367-rid23569143.denovo.08	100%	100%	ABVVCB010000008.1
149	*Acinetobacter baumannii*	Acinetobacter baumannii strain 2024HL-01114 isolate IDR2400056718-01-00 SAMN44099366-rid23569153.denovo.11	100%	100%	ABVVCA010000011.1
150	*Acinetobacter baumannii*	Acinetobacter baumannii strain 2024HL-01106 isolate IDR2400055147-01-00 SAMN44099358-rid23569123.denovo.10	100%	100%	ABVVCD010000010.1
151	*Acinetobacter baumannii*	Acinetobacter baumannii strain 2024HL-01103 isolate IDR2400055051-01-00 SAMN44099355-rid23569183.denovo.10	100%	100%	ABVVBY010000010.1
152	*Acinetobacter baumannii*	Acinetobacter baumannii strain 2024HL-01091 isolate IDR2400054309-01-00 SAMN43999559-rid23524373.denovo.10	100%	100%	ABVPJW010000010.1
153	*Acinetobacter baumannii*	Acinetobacter baumannii strain 2024HL-01089 isolate IDR2400053360-01-00 SAMN43999557-rid23524393.denovo.11	100%	100%	ABVPJV010000011.1
154	*Acinetobacter baumannii*	Acinetobacter baumannii strain 2024HL-01087 isolate IDR2400053191-01-02 SAMN43999555-rid23524413.denovo.10	100%	100%	ABVPJS010000010.1
155	*Acinetobacter baumannii*	Acinetobacter baumannii strain 2024HL-01085 isolate IDR2400052328-01-00 SAMN43999553-rid23524283.denovo.50	100%	100%	ABVPKF010000047.1
156	*Acinetobacter baumannii*	Acinetobacter baumannii strain 2024HL-01078 isolate IDR2400048397-01-00 SAMN43999546-rid23524353.denovo.12	100%	100%	ABVPJY010000012.1
157	*Acinetobacter baumannii*	Acinetobacter baumannii strain 2024HL-01072 isolate IDR2400055154-01-00 SAMN43944384-rid23467823.denovo.10	100%	100%	ABVEVE010000010.1
158	*Acinetobacter baumannii*	Acinetobacter baumannii strain 2024HL-01058 isolate IDR2400052457-01-00 SAMN43896550-rid23437863.denovo.09	100%	100%	ABUWAT010000009.1
159	*Acinetobacter baumannii*	Acinetobacter baumannii strain 2024HL-01053 isolate IDR2400051887-01-00 SAMN43896545-rid23437893.denovo.11	100%	100%	ABUWAP010000011.1
160	*Acinetobacter baumannii*	Acinetobacter baumannii strain 2024HL-01040 isolate IDR2400050569-01-00 SAMN43896532-rid23437803.denovo.12	100%	100%	ABUWBD010000012.1
161	*Acinetobacter baumannii*	Acinetobacter baumannii strain 2024HL-01038 isolate IDR2400050181-01-00 SAMN43896530-rid23437813.denovo.10	100%	100%	ABUWBC010000010.1
162	*Acinetobacter baumannii*	Acinetobacter baumannii strain 2024HL-01037 isolate IDR2400049999-01-02 SAMN43896529-rid23437823.denovo.08	100%	100%	ABUWBB010000008.1
163	*Acinetobacter baumannii*	Acinetobacter baumannii strain 2024HL-01036 isolate IDR2400049696-01-00 SAMN43896528-rid23437833.denovo.10	100%	100%	ABUWBA010000010.1
164	*Acinetobacter baumannii*	Acinetobacter baumannii strain 2024HL-01018 isolate IDR2400049007-01-00 SAMN43800974-rid23401423.denovo.10	100%	100%	ABUWQN010000010.1
165	*Acinetobacter baumannii*	Acinetobacter baumannii strain 2024HL-01017 isolate IDR2400049006-01-00 SAMN43800973-rid23401433.denovo.30	100%	100%	ABUWQM010000028.1
166	*Acinetobacter baumannii*	Acinetobacter baumannii strain 2024HL-01012 isolate IDR2400047711-01-04 SAMN43800968-rid23401523.denovo.10	100%	100%	ABUWQD010000010.1
167	*Acinetobacter baumannii*	Acinetobacter baumannii strain 2024HL-01011 isolate IDR2400047537-01-03 SAMN43800967-rid23401533.denovo.09	100%	100%	ABUWQA010000009.1
168	*Acinetobacter baumannii*	Acinetobacter baumannii strain 2024HL-01007 isolate IDR2400050840-01-00 SAMN43696438-rid23366433.denovo.33	100%	100%	ABUYCG010000029.1
169	*Acinetobacter baumannii*	Acinetobacter baumannii strain 2024HL-00999 isolate IDR2400048623-01-00 SAMN43696430-rid23366393.denovo.13	100%	100%	ABUYCK010000013.1
170	*Acinetobacter baumannii*	Acinetobacter baumannii strain 2024HL-00997 isolate IDR2400047876-01-00 SAMN43696428-rid23366403.denovo.09	100%	100%	ABUYCL010000009.1
171	*Acinetobacter baumannii*	Acinetobacter baumannii strain 2024HL-00996 isolate IDR2400047181-01-00 SAMN43696427-rid23366413.denovo.10	100%	100%	ABUYCH010000010.1
172	*Acinetobacter baumannii*	Acinetobacter baumannii strain 2024HL-00986 isolate IDR2400048398-01-00 SAMN43548133-rid23276183.denovo.03	100%	100%	ABVTPW010000003.1
173	*Acinetobacter baumannii*	Acinetobacter baumannii strain 2024HL-00984 isolate IDR2400048138-01-00 SAMN43548131-rid23278423.denovo.09	100%	100%	ABVTPY010000009.1
174	*Acinetobacter baumannii*	Acinetobacter baumannii strain 2024HL-00980 isolate IDR2400047180-01-00 SAMN43548127-rid23278413.denovo.09	100%	100%	ABVTQD010000009.1
175	*Acinetobacter baumannii*	Acinetobacter baumannii strain 2024HL-00974 isolate IDR2400045062-01-01 SAMN43548121-rid23278433.denovo.008	100%	100%	ABVTPU010000008.1
176	*Acinetobacter baumannii*	Acinetobacter baumannii strain 2024HL-00970 isolate IDR2400046966-01-02 SAMN43514800-rid23210233.denovo.11	100%	100%	ABULVH010000011.1
177	*Acinetobacter baumannii*	Acinetobacter baumannii strain 2024HL-00969 isolate IDR2400046959-01-02 SAMN43514799-rid23209153.denovo.13	100%	100%	ABULVD010000013.1
178	*Acinetobacter baumannii*	Acinetobacter baumannii strain 2024HL-00968 isolate IDR2400046870-01-00 SAMN43514798-rid23210243.denovo.10	100%	100%	ABULVF010000010.1
179	*Acinetobacter baumannii*	Acinetobacter baumannii strain 2024HL-00960 isolate IDR2400045847-01-02 SAMN43514790-rid23209113.denovo.10	100%	100%	ABULVK010000010.1
180	*Acinetobacter baumannii*	Acinetobacter baumannii strain 2024HL-00959 isolate IDR2400045185-01-03 SAMN43514789-rid23210203.denovo.10	100%	100%	ABULVL010000010.1
181	*Acinetobacter baumannii*	Acinetobacter baumannii strain 2024HL-00955 isolate IDR2400029032-01-00 SAMN43514785-rid23210263.denovo.09	100%	100%	ABULVB010000009.1
182	*Acinetobacter baumannii*	Acinetobacter baumannii strain 2024HL-00877 isolate IDR2400047185-01-00 SAMN43409815-rid23041953.denovo.09	100%	100%	ABUJMK010000009.1
183	*Acinetobacter baumannii*	Acinetobacter baumannii strain 2024HL-00873 isolate IDR2400046109-01-00 SAMN43409811-rid23041993.denovo.10	100%	100%	ABUJLG010000010.1
184	*Acinetobacter baumannii*	Acinetobacter baumannii strain 2024HL-00870 isolate IDR2400045270-01-00 SAMN43409808-rid23041893.denovo.09	100%	100%	ABUJMS010000009.1
185	*Acinetobacter baumannii*	Acinetobacter baumannii strain 2024HL-00869 isolate IDR2400045264-01-00 SAMN43409807-rid23041903.denovo.13	100%	100%	ABUJMU010000013.1
186	*Acinetobacter baumannii*	Acinetobacter baumannii strain 2024HL-00868 isolate IDR2400045263-02-00 SAMN43409806-rid23041913.denovo.09	100%	100%	ABUJMW010000009.1
187	*Acinetobacter baumannii*	Acinetobacter baumannii strain 2024HL-00853 isolate IDR2400044510-01-00 SAMN43227491-rid22887743.denovo.09	100%	100%	ABUDHK010000009.1
188	*Acinetobacter baumannii*	Acinetobacter baumannii strain 2024HL-00847 isolate IDR2400044185-01-00 SAMN43227485-rid22887803.denovo.31	100%	100%	ABUDHF010000027.1
189	*Acinetobacter baumannii*	Acinetobacter baumannii strain 2024HL-00844 isolate IDR2400043819-01-03 SAMN43227482-rid22887833.denovo.12	100%	100%	ABUDHB010000012.1
190	*Acinetobacter baumannii*	Acinetobacter baumannii strain 2024HL-00843 isolate IDR2400043337-01-00 SAMN43227481-rid22887603.denovo.09	100%	100%	ABUDHX010000009.1
191	*Acinetobacter baumannii*	Acinetobacter baumannii strain 2024HL-00842 isolate IDR2400043333-01-00 SAMN43227480-rid22887613.denovo.09	100%	100%	ABUDHY010000009.1
192	*Acinetobacter baumannii*	Acinetobacter baumannii strain 2024HL-00841 isolate IDR2400043318-01-00 SAMN43227479-rid22887623.denovo.11	100%	100%	ABUDHW010000011.1
193	*Acinetobacter baumannii*	Acinetobacter baumannii strain 2024HL-00840 isolate IDR2400043273-01-00 SAMN43227478-rid22887633.denovo.10	100%	100%	ABUDHV010000010.1
194	*Acinetobacter baumannii*	Acinetobacter baumannii strain 2024HL-00839 isolate IDR2400043195-01-00 SAMN43227477-rid22887643.denovo.10	100%	100%	ABUDHU010000010.1
195	*Acinetobacter baumannii*	Acinetobacter baumannii strain 2024HL-00830 isolate IDR2400042828-01-00 SAMN43163184-rid22818923.denovo.08	100%	100%	ABUBAJ010000008.1
196	*Acinetobacter baumannii*	Acinetobacter baumannii strain 2024HL-00825 isolate IDR2400041452-01-00 SAMN43163179-rid22818983.denovo.012	100%	100%	ABUAQG010000012.1
197	*Acinetobacter baumannii*	Acinetobacter baumannii strain 2024HL-00820 isolate IDR2400041066-01-00 SAMN43163174-rid22819033.denovo.008	100%	100%	ABUAQC010000008.1
198	*Acinetobacter baumannii*	Acinetobacter baumannii strain 2024HL-00806 isolate IDR2400042857-01-00 SAMN43160954-rid22816283.denovo.11	100%	100%	ABUAMA010000011.1
199	*Acinetobacter baumannii*	Acinetobacter baumannii strain 2024HL-00803 isolate IDR2400041957-01-02 SAMN43160951-rid22816293.denovo.53	100%	100%	ABUALY010000047.1
200	*Acinetobacter baumannii*	Acinetobacter baumannii strain 2024HL-00788 isolate IDR2400040815-01-00 SAMN43035890-rid22606303.denovo.10	100%	100%	ABTVTD010000010.1
201	*Acinetobacter baumannii*	Acinetobacter baumannii strain 2024HL-00784 isolate IDR2400040201-01-00 SAMN43035886-rid22608203.denovo.10	100%	100%	ABTVUT010000010.1
202	*Acinetobacter baumannii*	Acinetobacter baumannii strain 2024HL-00782 isolate IDR2400039801-01-00 SAMN43035884-rid22606253.denovo.34	100%	100%	ABTVSJ010000030.1
203	*Acinetobacter baumannii*	Acinetobacter baumannii strain 2024HL-00781 isolate IDR2400039271-01-00 SAMN43035883-rid22606263.denovo.09	100%	100%	ABTVTG010000009.1
204	*Acinetobacter baumannii*	Acinetobacter baumannii strain 2024HL-00778 isolate IDR2400039018-01-00 SAMN43035880-rid22606283.denovo.09	100%	100%	ABTVSE010000009.1
205	*Acinetobacter baumannii*	Acinetobacter baumannii strain 2024HL-00776 isolate IDR2400038890-01-00 SAMN43035878-rid22606293.denovo.05	100%	100%	ABTVXC010000005.1
206	*Acinetobacter baumannii*	Acinetobacter baumannii strain 2024HL-00775 isolate IDR2400038889-01-00 SAMN43035877-rid22608193.denovo.008	100%	100%	ABTVTC010000008.1
207	*Acinetobacter baumannii*	Acinetobacter baumannii strain 2024HL-00765 isolate IDR2400038283-01-00 SAMN42924603-rid22498213.denovo.10	100%	100%	ABTTQV010000010.1
208	*Acinetobacter baumannii*	Acinetobacter baumannii strain 2024HL-00763 isolate IDR2400038078-01-00 SAMN42924601-rid22498233.denovo.11	100%	100%	ABTTNA010000011.1
209	*Acinetobacter baumannii*	Acinetobacter baumannii strain 2024HL-00751 isolate IDR2400034271-01-03 SAMN42924589-rid22498273.denovo.08	100%	100%	ABTTNM010000008.1
210	*Acinetobacter baumannii*	Acinetobacter baumannii strain 2024HL-00729 isolate IDR2400034275-01-02 SAMN42920173-rid22496463.denovo.06	100%	100%	ABTTJT010000006.1
211	*Acinetobacter baumannii*	Acinetobacter baumannii strain 2024HL-00727 isolate IDR2400034273-01-03 SAMN42920171-rid22496573.denovo.13	100%	100%	ABTTJO010000013.1
212	*Acinetobacter baumannii*	Acinetobacter baumannii strain 2024HL-00724 isolate IDR2400037048-01-00 SAMN42761231-rid22392053.denovo.45	100%	100%	ABTPYP010000040.1
213	*Acinetobacter baumannii*	Acinetobacter baumannii strain 2024HL-00717 isolate IDR2400036442-01-00 SAMN42761224-rid22391013.denovo.50	100%	100%	ABTPTX010000044.1
214	*Acinetobacter baumannii*	Acinetobacter baumannii strain 2024HL-00715 isolate IDR2400036291-01-00 SAMN42761222-rid22391023.denovo.10	100%	100%	ABTQAL010000010.1
215	*Acinetobacter baumannii*	Acinetobacter baumannii strain 2024HL-00713 isolate IDR2400035751-01-00 SAMN42761220-rid22391033.denovo.10	100%	100%	ABTPYQ010000010.1
216	*Acinetobacter baumannii*	Acinetobacter baumannii strain 2024HL-00712 isolate IDR2400034883-01-00 SAMN42761219-rid22392033.denovo.09	100%	100%	ABTPZH010000009.1
217	*Acinetobacter baumannii*	Acinetobacter baumannii strain 2024HL-00705 isolate IDR2400034164-01-00 SAMN42761212-rid22390983.denovo.10	100%	100%	ABTPZG010000010.1
218	*Acinetobacter baumannii*	Acinetobacter baumannii strain 2024HL-00669 isolate IDR2400033073-01-01 SAMN42232250-rid22238573.denovo.09	100%	100%	ABTGSH010000009.1
219	*Acinetobacter baumannii*	Acinetobacter baumannii strain 2024HL-00659 isolate IDR2400033655-01-00 SAMN42089592-rid22186793.denovo.08	100%	100%	ABTDNP010000008.1
220	*Acinetobacter baumannii*	Acinetobacter baumannii strain 2024HL-00656 isolate IDR2400033163-01-00 SAMN42089589-rid22186813.denovo.10	100%	100%	ABTDNN010000010.1
221	*Acinetobacter baumannii*	Acinetobacter baumannii strain 2024HL-00655 isolate IDR2400033161-01-00 SAMN42089588-rid22186823.denovo.12	100%	100%	ABTDNM010000012.1
222	*Acinetobacter baumannii*	Acinetobacter baumannii strain 2024HL-00645 isolate IDR2400031957-01-00 SAMN42089578-rid22194483.denovo.012	100%	100%	ABTDUZ010000012.1
223	*Acinetobacter baumannii*	Acinetobacter baumannii strain 2024HL-00633 isolate IDR2400030893-01-00 SAMN42024193-rid22152763.denovo.10	100%	100%	ABTBUZ010000010.1
224	*Acinetobacter baumannii*	Acinetobacter baumannii strain 2024HL-00620 isolate IDR2400031177-01-00 SAMN42011110-rid22148743.denovo.10	100%	100%	ABTBNE010000010.1
225	*Acinetobacter baumannii*	Acinetobacter baumannii strain 2024HL-00617 isolate IDR2400031174-01-00 SAMN42011107-rid22137313.denovo.21	100%	100%	ABTBEI010000018.1
226	*Acinetobacter baumannii*	Acinetobacter baumannii strain 2024HL-00582 isolate IDR2400029511-01-00 SAMN41834509-rid21964573.denovo.008	100%	100%	ABSYOP010000008.1
227	*Acinetobacter baumannii*	Acinetobacter baumannii strain 2024HL-00576 isolate IDR2400028058-01-00 SAMN41834503-rid21965143.denovo.09	100%	100%	ABSYOR010000009.1
228	*Acinetobacter baumannii*	Acinetobacter baumannii strain 2024HL-00572 isolate IDR2400027531-01-00 SAMN41805236-rid21953823.denovo.11	100%	100%	ABSYKP010000011.1
229	*Acinetobacter baumannii*	Acinetobacter baumannii strain 2024HL-00571 isolate IDR2400027530-01-00 SAMN41805235-rid21959213.denovo.10	100%	100%	ABSYOC010000010.1
230	*Acinetobacter baumannii*	Acinetobacter baumannii strain 2024HL-00565 isolate IDR2400026699-01-02 SAMN41805229-rid21959323.denovo.10	100%	100%	ABSYNH010000010.1
231	*Acinetobacter baumannii*	Acinetobacter baumannii strain 2024HL-00540 isolate IDR2400026489-01-00 SAMN41612501-rid21782923.denovo.009	100%	100%	ABSRMI010000009.1
232	*Acinetobacter baumannii*	Acinetobacter baumannii strain 2024HL-00530 isolate IDR2400024986-01-00 SAMN41612491-rid21782933.denovo.10	100%	100%	ABSRMH010000010.1

233	*Acinetobacter baumannii*	Acinetobacter baumannii strain 2024HL-00527 isolate IDR2400026913-01-00 SAMN41526209-rid21714333.denovo.08	100%	100%	ABSLHR010000008.1
234	*Acinetobacter baumannii*	Acinetobacter baumannii strain 2024HL-00526 isolate IDR2400026911-01-00 SAMN41526208-rid21714343.denovo.08	100%	100%	ABSLGJ010000008.1
235	*Acinetobacter baumannii*	Acinetobacter baumannii strain 2024HL-00525 isolate IDR2400026910-01-00 SAMN41526207-rid21714353.denovo.10	100%	100%	ABSLHB010000010.1
236	*Acinetobacter baumannii*	Acinetobacter baumannii strain 2024HL-00512 isolate IDR2400024983-01-00 SAMN41396430-rid21621133.denovo.07	100%	100%	ABSEZB010000007.1
237	*Acinetobacter baumannii*	Acinetobacter baumannii strain 2024HL-00508 isolate IDR2400024698-01-00 SAMN41396426-rid21621173.denovo.11	100%	100%	ABSEYY010000011.1
238	*Acinetobacter baumannii*	Acinetobacter baumannii strain 2024HL-00507 isolate IDR2400024038-01-00 SAMN41396425-rid21621183.denovo.09	100%	100%	ABSEYZ010000009.1
239	*Acinetobacter baumannii*	Acinetobacter baumannii strain 2024HL-00501 isolate IDR2400023455-01-01 SAMN41396419-rid21621063.denovo.11	100%	100%	ABSEZH010000011.1
240	*Acinetobacter baumannii*	Acinetobacter baumannii strain 2024HL-00500 isolate IDR2400023439-01-00 SAMN41396418-rid21621073.denovo.08	100%	100%	ABSEZF010000008.1
241	*Acinetobacter baumannii*	Acinetobacter baumannii strain 2024HL-00499 isolate IDR2400023108-01-02 SAMN41396417-rid21621083.denovo.22	100%	100%	ABSEZG010000020.1
242	*Acinetobacter baumannii*	Acinetobacter baumannii strain 2024HL-00498 isolate IDR2400023016-01-02 SAMN41396416-rid21621093.denovo.09	100%	100%	ABSEZE010000009.1
243	*Acinetobacter baumannii*	Acinetobacter baumannii strain 2024HL-00495 isolate IDR2400022304-01-00 SAMN41396413-rid21621123.denovo.012	100%	100%	ABSKIP010000012.1
244	*Acinetobacter baumannii*	Acinetobacter baumannii strain 2024HL-00493 isolate IDR2400021077-01-00 SAMN41396411-rid21621243.denovo.53	100%	100%	ABSEYT010000047.1
245	*Acinetobacter baumannii*	Acinetobacter baumannii strain 2024HL-00475 isolate IDR2400021938-01-00 SAMN41190206-rid21558323.denovo.08	100%	100%	ABSAHW010000008.1
246	*Acinetobacter baumannii*	Acinetobacter baumannii strain 2024HL-00474 isolate IDR2400021746-01-00 SAMN41190205-rid21558333.denovo.09	100%	100%	ABSAHY010000009.1
247	*Acinetobacter baumannii*	Acinetobacter baumannii strain 2024HL-00414 isolate IDR2400019479-01-00 SAMN41059521-rid21486253.denovo.09	100%	100%	ABRWGC010000009.1
248	*Acinetobacter baumannii*	Acinetobacter baumannii strain 2024HL-00413 isolate IDR2400019457-01-00 SAMN41059520-rid21486263.denovo.12	100%	100%	ABRWFX010000012.1
249	*Acinetobacter baumannii*	Acinetobacter baumannii strain 2024HL-00398 isolate IDR2400013854-01-00 SAMN40982211-rid21450033.denovo.10	100%	100%	ABRTIJ010000010.1
250	*Acinetobacter baumannii*	Acinetobacter baumannii strain 2024HL-00393 isolate IDR2400013690-01-02 SAMN40982206-rid21450143.denovo.09	100%	100%	ABRTIW010000009.1
251	*Acinetobacter baumannii*	Acinetobacter baumannii strain 2024HL-00365 isolate IDR2400013678-01-02 SAMN40752280-rid21393653.denovo.10	100%	100%	ABROMJ010000010.1
252	*Acinetobacter baumannii*	Acinetobacter baumannii strain 2024HL-00311 isolate IDR2400011263-01-00 SAMN40556757-rid21284973.denovo.10	100%	100%	ABQURE010000010.1
253	*Acinetobacter baumannii*	Acinetobacter baumannii strain 2024HL-00310 isolate IDR2400011260-01-01 SAMN40556756-rid21284653.denovo.11	100%	100%	ABQUSK010000011.1
254	*Acinetobacter baumannii*	Acinetobacter baumannii strain 2024HL-00308 isolate IDR2400011257-01-00 SAMN40556754-rid21284873.denovo.08	100%	100%	ABQUSF010000008.1
255	*Acinetobacter baumannii*	Acinetobacter baumannii strain 2024HL-00283 isolate IDR2400008628-01-02 SAMN40545834-rid21276863.denovo.06	100%	100%	ABQZAI010000006.1
256	*Acinetobacter baumannii*	Acinetobacter baumannii strain 2024HL-00280 isolate IDR2400008609-01-02 SAMN40545831-rid21279593.denovo.09	100%	100%	ABQZAG010000009.1
257	*Acinetobacter baumannii*	Acinetobacter baumannii strain 2024HL-00222 isolate IDR2400006426-01-00 SAMN40376023-rid21228153.denovo.13	100%	100%	ABQQGN010000013.1
258	*Acinetobacter baumannii*	Acinetobacter baumannii strain 2024HL-00219 isolate IDR2400005804-01-00 SAMN40376020-rid21228183.denovo.08	100%	100%	ABQQGK010000008.1
259	*Acinetobacter baumannii*	Acinetobacter baumannii strain 2024HL-00148 isolate IDR2400002695-01-00 SAMN39856827-rid20936113.denovo.009	100%	100%	ABQDGM010000009.1
260	*Acinetobacter baumannii*	Acinetobacter baumannii strain 2024HL-00145 isolate IDR2400002479-01-00 SAMN39856824-rid20936143.denovo.12	100%	100%	ABQDDT010000012.1
261	*Acinetobacter baumannii*	Acinetobacter baumannii strain 2024HL-00123 isolate IDR2400001048-01-00 SAMN39832815-rid20862673.denovo.10	100%	100%	ABQCDW010000010.1
262	*Acinetobacter baumannii*	Acinetobacter baumannii strain 2024HL-00119 isolate IDR2400001035-01-00 SAMN39832811-rid20860803.denovo.07	100%	100%	ABQCEJ010000007.1
263	*Acinetobacter baumannii*	Acinetobacter baumannii strain 2024HL-00102 isolate IDR2300074378-01-00 SAMN39502252-rid20768533.denovo.10	100%	100%	ABPYBW010000010.1
264	*Acinetobacter baumannii*	Acinetobacter baumannii strain 2024HL-00101 isolate IDR2300073847-01-00 SAMN39502251-rid20768373.denovo.04	100%	100%	ABPYBV010000004.1
265	*Acinetobacter baumannii*	Acinetobacter baumannii strain 2024HL-00068 isolate IDR2300071798-01-00 SAMN39405749-rid20718863.denovo.10	100%	100%	ABPVGK010000010.1
266	*Acinetobacter baumannii*	Acinetobacter baumannii strain 2024HL-00066 isolate IDR2300071795-01-00 SAMN39405747-rid20719793.denovo.12	100%	100%	ABPVGI010000012.1
267	*Acinetobacter baumannii*	Acinetobacter baumannii strain 2024HL-00060 isolate IDR2300071160-01-00 SAMN39331327-rid20707893.denovo.12	100%	100%	ABPTTU010000012.1
268	*Acinetobacter baumannii*	Acinetobacter baumannii strain 2024HL-00059 isolate IDR2300071092-01-00 SAMN39331326-rid20707903.denovo.10	100%	100%	ABPTUB010000010.1
269	*Acinetobacter baumannii*	Acinetobacter baumannii strain 2024HL-00056 isolate IDR2300070854-01-00 SAMN39331323-rid20707933.denovo.13	100%	100%	ABPTWE010000013.1
270	*Acinetobacter baumannii*	Acinetobacter baumannii strain 2024HL-00032 isolate IDR2300069664-01-00 SAMN39313709-rid20704293.denovo.09	100%	100%	ABPTNY010000009.1
271	*Acinetobacter baumannii*	Acinetobacter baumannii strain 2024HL-00031 isolate IDR2300069596-01-00 SAMN39313708-rid20705263.denovo.08	100%	100%	ABPTNW010000008.1
272	*Acinetobacter baumannii*	Acinetobacter baumannii strain 2024HL-00028 isolate IDR2300069557-01-00 SAMN39313705-rid20703423.denovo.09	100%	100%	ABPTRE010000009.1
273	*Acinetobacter baumannii*	Acinetobacter baumannii strain 2024HL-00021 isolate IDR2300069194-01-00 SAMN39313698-rid20705303.denovo.10	100%	100%	ABPTNU010000010.1
274	*Acinetobacter baumannii*	Acinetobacter baumannii strain 2024HL-00020 isolate IDR2300069193-01-00 SAMN39313697-rid20704353.denovo.35	100%	100%	ABPTRC010000031.1
275	*Acinetobacter baumannii*	Acinetobacter baumannii strain 2024HL-00013 isolate IDR2300068329-01-00 SAMN39313690-rid20705213.denovo.012	100%	100%	ABPTPC010000012.1
276	*Acinetobacter baumannii*	Acinetobacter baumannii strain 2024HL-00010 isolate IDR2300068038-01-01 SAMN39313687-rid20705223.denovo.13	100%	100%	ABPUTR010000013.1
277	*Acinetobacter baumannii*	Acinetobacter baumannii strain 2024HL-00008 isolate IDR2300067518-01-02 SAMN39313685-rid20705233.denovo.09	100%	100%	ABPTNZ010000009.1
278	*Acinetobacter baumannii*	Acinetobacter baumannii strain 2024GO-0526 SAMN45207010-rid24447133.denovo.14	100%	100%	ABWQUF010000014.1
279	*Acinetobacter baumannii*	Acinetobacter baumannii strain 2024GO-0390 SAMN43384002-rid23021783.denovo.14	100%	100%	ABUIFF010000014.1
280	*Acinetobacter baumannii*	Acinetobacter baumannii strain 2024EP-00341 SAMN44309224-rid23633263.denovo.16	100%	100%	ABVXMA010000015.1
281	*Acinetobacter baumannii*	Acinetobacter baumannii strain 2024EP-00225 SAMN43035012-rid22603203.denovo.01	100%	100%	ABTVQR010000001.1
282	*Acinetobacter baumannii*	Acinetobacter baumannii strain 2024EP-00211 SAMN42749623-rid22389263.denovo.10	100%	100%	ABTPPW010000010.1
283	*Acinetobacter baumannii*	Acinetobacter baumannii strain 2024EP-00189 SAMN42156579-rid22212433.denovo.15	100%	100%	ABTFBN010000015.1
284	*Acinetobacter baumannii*	Acinetobacter baumannii strain 2023HL-01074 isolate IDR2300066855-01-00 SAMN39080910-rid20650183.denovo.11	100%	100%	ABPOMI010000011.1
285	*Acinetobacter baumannii*	Acinetobacter baumannii strain 2023HL-01073 isolate IDR2300066854-01-00 SAMN39080909-rid20650193.denovo.011	100%	100%	ABPOMH010000011.1
286	*Acinetobacter baumannii*	Acinetobacter baumannii strain 2023HL-01070 isolate IDR2300066094-01-02 SAMN39080906-rid20650223.denovo.11	100%	100%	ABPOMG010000011.1
287	*Acinetobacter baumannii*	Acinetobacter baumannii strain 2023HL-01054 isolate IDR2300064838-01-00 SAMN39080890-rid20650383.denovo.13	100%	100%	ABPOLP010000013.1
288	*Acinetobacter baumannii*	Acinetobacter baumannii strain 2023HL-01051 NODE_10_length_138020_cov_32.677823	100%	100%	ABPOLM020000010.1
289	*Acinetobacter baumannii*	Acinetobacter baumannii strain 2023HL-01050 isolate IDR2300062956-01-00 SAMN39080886-rid20650423.denovo.09	100%	100%	ABPOLL010000009.1
290	*Acinetobacter baumannii*	Acinetobacter baumannii strain 2023HL-01047 isolate IDR2300062926-01-00 SAMN39080883-rid20650113.denovo.09	100%	100%	ABPOMR010000009.1
291	*Acinetobacter baumannii*	Acinetobacter baumannii strain 2023HL-01045 isolate IDR2300062694-01-00 SAMN39080881-rid20650133.denovo.09	100%	100%	ABPOMQ010000009.1
292	*Acinetobacter baumannii*	Acinetobacter baumannii strain 2023HL-01044 isolate IDR2300062601-01-00 SAMN39080880-rid20650563.denovo.09	100%	100%	ABPOMN010000009.1
293	*Acinetobacter baumannii*	Acinetobacter baumannii strain 2023HL-01042 isolate IDR2300061891-01-02 SAMN39080878-rid20650233.denovo.10	100%	100%	ABPOME010000010.1
294	*Acinetobacter baumannii*	Acinetobacter baumannii strain 2023HL-01004 isolate IDR2300061639-01-02 SAMN38761678-rid20543843.denovo.10	100%	100%	ABPKEQ010000010.1
295	*Acinetobacter baumannii*	Acinetobacter baumannii strain 2023HL-00972 isolate IDR2300058333-01-02 SAMN38447755-rid20421893.denovo.08	100%	100%	ABPEGM010000008.1
296	*Acinetobacter baumannii*	Acinetobacter baumannii strain 2023HL-00967 isolate IDR2300061238-01-00 SAMN38447456-rid20420883.denovo.10	100%	100%	ABPECX010000010.1
297	*Acinetobacter baumannii*	Acinetobacter baumannii strain 2023HL-00966 isolate IDR2300061236-01-00 SAMN38447455-rid20420893.denovo.18	100%	100%	ABPEDG010000017.1
298	*Acinetobacter baumannii*	Acinetobacter baumannii strain 2023HL-00964 isolate IDR2300060640-01-00 SAMN38447453-rid20420913.denovo.52	100%	100%	ABPECI010000050.1
299	*Acinetobacter baumannii*	Acinetobacter baumannii strain 2023HL-00946 isolate IDR2300059920-01-01 SAMN38198384-rid20810913.denovo.01	100%	100%	ABQAWC010000001.1
300	*Acinetobacter baumannii*	Acinetobacter baumannii strain 2023HL-00945 isolate IDR2300059919-01-02 SAMN38198383-rid20810383.denovo.02	100%	100%	ABQAIN010000002.1
301	*Acinetobacter baumannii*	Acinetobacter baumannii strain 2023HL-00939 isolate IDR2300059494-01-00 SAMN38198377-rid20810423.denovo.10	100%	100%	ABQAIG010000010.1
302	*Acinetobacter baumannii*	Acinetobacter baumannii strain 2023HL-00926 isolate IDR2300057228-01-02 SAMN38198364-rid20810333.denovo.10	100%	100%	ABQAIK010000010.1
303	*Acinetobacter baumannii*	Acinetobacter baumannii strain 2023HL-00898 isolate IDR2300054490-01-02 SAMN38180886-rid20986743.denovo.10	100%	100%	ABRARW010000010.1
304	*Acinetobacter baumannii*	Acinetobacter baumannii strain 2023HL-00897 isolate IDR2300054487-01-02 SAMN38180885-rid20986753.denovo.10	100%	100%	ABQGMQ010000010.1
305	*Acinetobacter baumannii*	Acinetobacter baumannii strain 2023HL-00895 isolate IDR2300053431-01-02 SAMN38180883-rid20810223.denovo.29	100%	100%	ABQAIV010000027.1
306	*Acinetobacter baumannii*	Acinetobacter baumannii strain 2023HL-00892 isolate IDR2300053427-01-02 SAMN38180880-rid20809623.denovo.08	100%	100%	ABQAKF010000008.1
307	*Acinetobacter baumannii*	Acinetobacter baumannii strain 2023HL-00866 isolate IDR2300054927-01-00 SAMN38089878-rid20891173.denovo.10	100%	100%	ABOOPF020000010.1
308	*Acinetobacter baumannii*	Acinetobacter baumannii strain 2023HL-00850 isolate IDR2300056348-01-00 SAMN38035081-rid20927813.denovo.12	100%	100%	ABOKSD020000012.1
309	*Acinetobacter baumannii*	Acinetobacter baumannii strain 2023HL-00837 isolate IDR2300051767-01-04 SAMN38035068-rid20940903.denovo.09	100%	100%	ABOKSF020000009.1
310	*Acinetobacter baumannii*	Acinetobacter baumannii strain 2023HL-00836 isolate IDR2300051766-01-02 SAMN38035067-rid20903373.denovo.10	100%	100%	ABOKSR020000010.1
311	*Acinetobacter baumannii*	Acinetobacter baumannii strain 2023HL-00815 isolate IDR2300052588-01-01 SAMN37806924-rid20909443.denovo.10	100%	100%	ABNVTD020000010.1
312	*Acinetobacter baumannii*	Acinetobacter baumannii strain 2023HL-00798 isolate IDR2300051120-01-01 SAMN37748406-rid20868353.denovo.10	100%	100%	ABNROA020000010.1
313	*Acinetobacter baumannii*	Acinetobacter baumannii strain 2023HL-00753 isolate IDR2300048550-01-02 SAMN37528599-rid20881073.denovo.10	100%	100%	ABMWBQ020000010.1
314	*Acinetobacter baumannii*	Acinetobacter baumannii strain 2023HL-00752 isolate IDR2300048548-01-02 SAMN37528598-rid20883803.denovo.10	100%	100%	ABMWFF020000010.1
315	*Acinetobacter baumannii*	Acinetobacter baumannii strain 2023HL-00751 isolate IDR2300048386-01-00 SAMN37528597-rid20882553.denovo.09	100%	100%	ABMWFL020000009.1
316	*Acinetobacter baumannii*	Acinetobacter baumannii strain 2023HL-00750 isolate IDR2300048378-01-00 SAMN37528596-rid20904463.denovo.10	100%	100%	ABMWDM020000010.1
317	*Acinetobacter baumannii*	Acinetobacter baumannii strain 2023HL-00748 isolate IDR2300048346-01-00 SAMN37528594-rid20907973.denovo.30	100%	100%	ABMWBZ020000026.1
318	*Acinetobacter baumannii*	Acinetobacter baumannii strain 2023HL-00744 isolate IDR2300048135-01-02 SAMN37528590-rid20892313.denovo.10	100%	100%	ABMWBX020000010.1
319	*Acinetobacter baumannii*	Acinetobacter baumannii strain 2023HL-00735 isolate IDR2300046861-01-00 SAMN37513285-rid20941913.denovo.13	100%	100%	ABMUZM020000013.1
320	*Acinetobacter baumannii*	Acinetobacter baumannii strain 2023HL-00729 isolate IDR2300046707-01-01 SAMN37513279-rid20944933.denovo.10	100%	100%	ABMUZW020000010.1
321	*Acinetobacter baumannii*	Acinetobacter baumannii strain 2023HL-00727 isolate IDR2300046569-01-00 SAMN37513277-rid20947463.denovo.09	100%	100%	ABMUZU020000009.1
322	*Acinetobacter baumannii*	Acinetobacter baumannii strain 2023HL-00726 isolate IDR2300045767-01-02 SAMN37513276-rid20882543.denovo.08	100%	100%	ABMUZS020000008.1
323	*Acinetobacter baumannii*	Acinetobacter baumannii strain 2023HL-00724 isolate IDR2300041710-01-00 SAMN37513274-rid20898453.denovo.09	100%	100%	ABMUZK020000009.1
324	*Acinetobacter baumannii*	Acinetobacter baumannii strain 2023HL-00706 isolate IDR2300045578-01-02 SAMN37327923-rid20900283.denovo.10	100%	100%	ABMDUF020000010.1
325	*Acinetobacter baumannii*	Acinetobacter baumannii strain 2023HL-00702 isolate IDR2300041780-01-02 SAMN37327919-rid20909333.denovo.10	100%	100%	ABMDUB020000010.1
326	*Acinetobacter baumannii*	Acinetobacter baumannii strain 2023HL-00701 isolate IDR2300040900-01-02 SAMN37327918-rid20939853.denovo.12	100%	100%	ABMDUA020000012.1
327	*Acinetobacter baumannii*	Acinetobacter baumannii strain 2023HL-00700 isolate IDR2300040899-01-03 SAMN37327917-rid20926573.denovo.09	100%	100%	ABMDUN020000009.1
328	*Acinetobacter baumannii*	Acinetobacter baumannii strain 2023HL-00699 isolate IDR2300040898-01-05 SAMN37327916-rid20898433.denovo.12	100%	100%	ABMDUK020000012.1
329	*Acinetobacter baumannii*	Acinetobacter baumannii strain 2023HL-00696 isolate IDR2300040896-01-02 SAMN37327913-rid20878443.denovo.09	100%	100%	ABMDUJ020000009.1
330	*Acinetobacter baumannii*	Acinetobacter baumannii strain 2023HL-00694 isolate IDR2300040894-01-02 SAMN37327911-rid20883773.denovo.10	100%	100%	ABMDUH020000010.1
331	*Acinetobacter baumannii*	Acinetobacter baumannii strain 2023HL-00693 isolate IDR2300040893-01-03 SAMN37327910-rid20902763.denovo.08	100%	100%	ABMDUG020000008.1
332	*Acinetobacter baumannii*	Acinetobacter baumannii strain 2023HL-00692 isolate IDR2300040892-01-02 SAMN37327909-rid20903353.denovo.09	100%	100%	ABMDTZ020000009.1
333	*Acinetobacter baumannii*	Acinetobacter baumannii strain 2023HL-00679 isolate IDR2300045430-01-00 SAMN37314359-rid20881053.denovo.13	100%	100%	ABMDHS020000013.1
334	*Acinetobacter baumannii*	Acinetobacter baumannii strain 2023HL-00660 SAMN37299132-rid20940813.denovo.09	100%	100%	ABMCCE020000009.1
335	*Acinetobacter baumannii*	Acinetobacter baumannii strain 2023HL-00651 SAMN37299123-rid20871183.denovo.09	100%	100%	ABMCCM020000009.1
336	*Acinetobacter baumannii*	Acinetobacter baumannii strain 2023HL-00639 SAMN37144225-rid20940223.denovo.10	100%	100%	ABLYKJ020000010.1
337	*Acinetobacter baumannii*	Acinetobacter baumannii strain 2023HL-00628 SAMN37144214-rid20878833.denovo.10	100%	100%	ABLYKI020000010.1
338	*Acinetobacter baumannii*	Acinetobacter baumannii strain 2023HL-00620 SAMN37111205-rid20899643.denovo.10	100%	100%	ABLYKW020000010.1
339	*Acinetobacter baumannii*	Acinetobacter baumannii strain 2023HL-00605 SAMN37001181-rid20881013.denovo.12	100%	100%	ABLYNC020000012.1
340	*Acinetobacter baumannii*	Acinetobacter baumannii strain 2023HL-00601 SAMN37001177-rid20879983.denovo.09	100%	100%	ABLYNJ020000009.1
341	*Acinetobacter baumannii*	Acinetobacter baumannii strain 2023HL-00579 SAMN36905497-rid20949873.denovo.10	100%	100%	ABLYQC020000010.1
342	*Acinetobacter baumannii*	Acinetobacter baumannii strain 2023HL-00575 NODE_12_length_135660_cov_54.154289	100%	100%	ABLYQB030000012.1
343	*Acinetobacter baumannii*	Acinetobacter baumannii strain 2023HL-00574 SAMN36905492-rid20904363.denovo.10	100%	100%	ABLYPZ020000010.1
344	*Acinetobacter baumannii*	Acinetobacter baumannii strain 2023HL-00569 SAMN36799761-rid20939783.denovo.029	100%	100%	ABLYSZ020000027.1
345	*Acinetobacter baumannii*	Acinetobacter baumannii strain 2023HL-00566 SAMN36781178-rid20878383.denovo.16	100%	100%	ABOMTX020000015.1
346	*Acinetobacter baumannii*	Acinetobacter baumannii strain 2023HL-00563 SAMN36781175-rid20941813.denovo.10	100%	100%	ABLYTL020000010.1
347	*Acinetobacter baumannii*	Acinetobacter baumannii strain 2023HL-00551 SAMN36781163-rid20937223.denovo.10	100%	100%	ABLYTM020000010.1
348	*Acinetobacter baumannii*	Acinetobacter baumannii strain 2023HL-00537 SAMN36708967-rid20939753.denovo.18	100%	100%	ABLYVR020000017.1
349	*Acinetobacter baumannii*	Acinetobacter baumannii strain 2023HL-00527 SAMN36708957-rid20880963.denovo.03	100%	100%	ABLYVU020000003.1

350	*Acinetobacter baumannii*	Acinetobacter baumannii strain 2023HL-00526 SAMN36708956-rid20907793.denovo.09	100%	100%	ABLYVP020000009.1
351	*Acinetobacter baumannii*	Acinetobacter baumannii strain 2023HL-00523 SAMN36458818-rid20892213.denovo.10	100%	100%	ABLYZY020000010.1
352	*Acinetobacter baumannii*	Acinetobacter baumannii strain 2023HL-00522 SAMN36458817-rid20871943.denovo.10	100%	100%	ABLYZX020000010.1
353	*Acinetobacter baumannii*	Acinetobacter baumannii strain 2023HL-00519 SAMN36458814-rid20900253.denovo.06	100%	100%	ABLYZV020000006.1
354	*Acinetobacter baumannii*	Acinetobacter baumannii strain 2023HL-00518 SAMN36458813-rid20880933.denovo.06	100%	100%	ABLYZU020000006.1
355	*Acinetobacter baumannii*	Acinetobacter baumannii strain 2023HL-00503 SAMN36340099-rid20941173.denovo.12	100%	100%	ABLZEH020000012.1
356	*Acinetobacter baumannii*	Acinetobacter baumannii strain 2023HL-00502 SAMN36340098-rid20939723.denovo.10	100%	100%	ABLZEG020000010.1
357	*Acinetobacter baumannii*	Acinetobacter baumannii strain 2023HL-00501 SAMN36340097-rid20941743.denovo.08	100%	100%	ABLZEP020000008.1
358	*Acinetobacter baumannii*	Acinetobacter baumannii strain 2023HL-00500 SAMN36340096-rid20893283.denovo.09	100%	100%	ABLZEQ020000009.1
359	*Acinetobacter baumannii*	Acinetobacter baumannii strain 2023HL-00499 SAMN36340095-rid20930563.denovo.11	100%	100%	ABLZEO020000011.1
360	*Acinetobacter baumannii*	Acinetobacter baumannii strain 2023HL-00498 SAMN36340094-rid20937163.denovo.08	100%	100%	ABLZEN020000008.1
361	*Acinetobacter baumannii*	Acinetobacter baumannii strain 2023HL-00495 SAMN36340091-rid20909243.denovo.10	100%	100%	ABLZEL020000010.1
362	*Acinetobacter baumannii*	Acinetobacter baumannii strain 2023HL-00494 NODE_11_length_135660_cov_29.441604	100%	100%	ABLZEK030000011.1
363	*Acinetobacter baumannii*	Acinetobacter baumannii strain 2023HL-00487 SAMN36019605-rid20866403.denovo.10	100%	100%	ABLZHA020000010.1
364	*Acinetobacter baumannii*	Acinetobacter baumannii strain 2023HL-00485 SAMN36019603-rid20927143.denovo.10	100%	100%	ABLZGX020000010.1
365	*Acinetobacter baumannii*	Acinetobacter baumannii strain 2023HL-00469 SAMN36019587-rid20868143.denovo.10	100%	100%	ABLZHD020000010.1
366	*Acinetobacter baumannii*	Acinetobacter baumannii strain 2023HL-00461 SAMN36019579-rid20899563.denovo.10	100%	100%	ABLZGO020000010.1
367	*Acinetobacter baumannii*	Acinetobacter baumannii strain 2023HL-00452 SAMN35996133-rid20939713.denovo.09	100%	100%	ABLZHJ020000009.1
368	*Acinetobacter baumannii*	Acinetobacter baumannii strain 2023HL-00450 SAMN35996131-rid20895963.denovo.12	100%	100%	ABLZHN020000012.1
369	*Acinetobacter baumannii*	Acinetobacter baumannii strain 2023HL-00446 SAMN35996127-rid20926423.denovo.09	100%	100%	ABLZHL020000009.1
370	*Acinetobacter baumannii*	Acinetobacter baumannii strain 2023HL-00440 SAMN35733029-rid20941713.denovo.13	100%	100%	ABLZND020000013.1
371	*Acinetobacter baumannii*	Acinetobacter baumannii strain 2023HL-00438 SAMN35733027-rid20864953.denovo.10	100%	100%	ABLZNA020000010.1
372	*Acinetobacter baumannii*	Acinetobacter baumannii strain 2023HL-00429 SAMN35733018-rid20868763.denovo.10	100%	100%	ABLZMR020000010.1
373	*Acinetobacter baumannii*	Acinetobacter baumannii strain 2023HL-00428 SAMN35733017-rid20866993.denovo.10	100%	100%	ABLZMQ020000010.1
374	*Acinetobacter baumannii*	Acinetobacter baumannii strain 2023HL-00427 SAMN35733016-rid20878323.denovo.09	100%	100%	ABLZMP020000009.1
375	*Acinetobacter baumannii*	Acinetobacter baumannii strain 2023HL-00426 SAMN35733015-rid20897253.denovo.11	100%	100%	ABLZNG020000011.1
376	*Acinetobacter baumannii*	Acinetobacter baumannii strain 2023HL-00420 SAMN35733009-rid20868133.denovo.11	100%	100%	ABLZNC020000011.1
377	*Acinetobacter baumannii*	Acinetobacter baumannii strain 2023HL-00417 SAMN35733006-rid20939703.denovo.10	100%	100%	ABLZMM020000010.1
378	*Acinetobacter baumannii*	Acinetobacter baumannii strain 2023HL-00414 SAMN35540152-rid20944183.denovo.10	100%	100%	ABMAEI020000010.1
379	*Acinetobacter baumannii*	Acinetobacter baumannii strain 2023HL-00357 SAMN35009974-rid20938643.denovo.05	100%	100%	ABLZPR020000005.1
380	*Acinetobacter baumannii*	Acinetobacter baumannii strain 2023HL-00330 SAMN34352519-rid21206063.denovo.09	100%	100%	ABLZXG030000009.1
381	*Acinetobacter baumannii*	Acinetobacter baumannii strain 2023HL-00280 SAMN34114927-rid21206293.denovo.09	100%	100%	ABLKIK040000009.1
382	*Acinetobacter baumannii*	Acinetobacter baumannii strain 2023HL-00278 SAMN34114925-rid21206223.denovo.10	100%	100%	ABLKIM040000010.1
383	*Acinetobacter baumannii*	Acinetobacter baumannii strain 2023HL-00276 SAMN34114923-rid21206233.denovo.09	100%	100%	ABLKIN040000009.1
384	*Acinetobacter baumannii*	Acinetobacter baumannii strain 2023HL-00271 SAMN34114918-rid21206273.denovo.13	100%	100%	ABLKIL040000013.1
385	*Acinetobacter baumannii*	Acinetobacter baumannii strain 2023HL-00048 SAMN33222307-rid20899293.denovo.47	100%	100%	ABKOFO030000043.1
386	*Acinetobacter baumannii*	Acinetobacter baumannii strain 2023HL-00041 SAMN33222300-rid20907233.denovo.10	100%	100%	ABKOGF030000010.1
387	*Acinetobacter baumannii*	Acinetobacter baumannii strain 2023HL-00008 SAMN32908113-rid20634643.denovo.09	100%	100%	ABKIWM030000009.1
388	*Acinetobacter baumannii*	Acinetobacter baumannii strain 2023GO-0402 SAMN38266322-rid20226743.denovo.06	100%	100%	ABOYJX010000006.1
389	*Acinetobacter baumannii*	Acinetobacter baumannii strain 2023GO-0340 SAMN38040988-rid20872043.denovo.10	100%	100%	ABOLDL020000010.1
390	*Acinetobacter baumannii*	Acinetobacter baumannii strain 2023EP-00177 SAMN37094441-rid20894583.denovo.11	100%	100%	ABLYMA020000011.1
391	*Acinetobacter baumannii*	Acinetobacter baumannii strain 2022HL-01983 SAMN32801557-rid20884523.denovo.10	100%	100%	ABKFYD030000010.1
392	*Acinetobacter baumannii*	Acinetobacter baumannii strain 2022HL-01982 SAMN32801556-rid20937013.denovo.11	100%	100%	ABKFYC030000011.1
393	*Acinetobacter baumannii*	Acinetobacter baumannii strain 2022HL-01970 SAMN32372806-rid20905283.denovo.11	100%	100%	ABJYTP030000011.1
394	*Acinetobacter baumannii*	Acinetobacter baumannii strain 2022HL-01902 SAMN31854458-rid20914863.denovo.09	100%	100%	ABJNMR030000009.1
395	*Acinetobacter baumannii*	Acinetobacter baumannii strain 2022HL-01900 SAMN31854456-rid20908863.denovo.07	100%	100%	ABJNMP030000007.1
396	*Acinetobacter baumannii*	Acinetobacter baumannii strain 2022HL-01899 SAMN31854455-rid20891943.denovo.09	100%	100%	ABJNMO030000009.1
397	*Acinetobacter baumannii*	Acinetobacter baumannii strain 2022HL-01894 SAMN31854450-rid20941303.denovo.09	100%	100%	ABJNME030000009.1
398	*Acinetobacter baumannii*	Acinetobacter baumannii strain 2022HL-01893 SAMN31854449-rid20903143.denovo.10	100%	100%	ABJNMF030000010.1
399	*Acinetobacter baumannii*	Acinetobacter baumannii strain 2022HL-01888 SAMN31854209-rid20913423.denovo.10	100%	100%	ABJNNL030000010.1
400	*Acinetobacter baumannii*	Acinetobacter baumannii strain 2022HL-01887 SAMN31854208-rid20899213.denovo.10	100%	100%	ABJNNJ030000010.1
401	*Acinetobacter baumannii*	Acinetobacter baumannii strain 2022HL-01885 SAMN31854206-rid20635273.denovo.03	100%	100%	ABJNNI030000003.1
402	*Acinetobacter baumannii*	Acinetobacter baumannii strain 2022HL-01874 SAMN31854195-rid20938533.denovo.10	100%	100%	ABJNNF030000010.1
403	*Acinetobacter baumannii*	Acinetobacter baumannii strain 2022HL-01873 SAMN31854194-rid20634443.denovo.49	100%	100%	ABJNNC030000043.1
404	*Acinetobacter baumannii*	Acinetobacter baumannii strain 2022HL-01870 SAMN31854157-rid20879283.denovo.10	100%	100%	ABJNDI030000010.1
405	*Acinetobacter baumannii*	Acinetobacter baumannii strain 2022HL-01830 SAMN31384863-rid20947113.denovo.13	100%	100%	ABIXSX030000013.1
406	*Acinetobacter baumannii*	Acinetobacter baumannii strain 2022HL-01825 SAMN31384858-rid20831923.denovo.09	100%	100%	ABIXSH030000009.1
407	*Acinetobacter baumannii*	Acinetobacter baumannii strain 2022HL-01765 SAMN30889467-rid20879043.denovo.10	100%	100%	ABIHPX030000010.1
408	*Acinetobacter baumannii*	Acinetobacter baumannii strain 2022HL-01764 SAMN30889466-rid20891873.denovo.10	100%	100%	ABIHOY030000010.1
409	*Acinetobacter baumannii*	Acinetobacter baumannii strain 2022HL-01763 SAMN30889465-rid20880343.denovo.10	100%	100%	ABIHOZ030000010.1
410	*Acinetobacter baumannii*	Acinetobacter baumannii strain 2022HL-01749 SAMN30889320-rid20896973.denovo.08	100%	100%	ABIIQB030000008.1
411	*Acinetobacter baumannii*	Acinetobacter baumannii strain 2022HL-01748 SAMN30889319-rid20926043.denovo.08	100%	100%	ABIHRK030000008.1
412	*Acinetobacter baumannii*	Acinetobacter baumannii strain 2022HL-01747 SAMN30889318-rid20941533.denovo.08	100%	100%	ABIHOS030000008.1
413	*Acinetobacter baumannii*	Acinetobacter baumannii strain 2022HL-01740 SAMN30889311-rid20922223.denovo.07	100%	100%	ABIHOD030000007.1
414	*Acinetobacter baumannii*	Acinetobacter baumannii strain 2022HL-01731 SAMN30889275-rid20913373.denovo.10	100%	100%	ABIHRX030000010.1
415	*Acinetobacter baumannii*	Acinetobacter baumannii strain 2022HL-01722 SAMN30889266-rid20943403.denovo.09	100%	100%	ABIIQL030000009.1
416	*Acinetobacter baumannii*	Acinetobacter baumannii strain 2022HL-01713 SAMN30889245-rid20900573.denovo.10	100%	100%	ABIIQR030000010.1
417	*Acinetobacter baumannii*	Acinetobacter baumannii strain 2022HL-01697 SAMN30889203-rid20877933.denovo.08	100%	100%	ABIHOU030000008.1
418	*Acinetobacter baumannii*	Acinetobacter baumannii strain 2022HL-01692 SAMN30889198-rid20914753.denovo.05	100%	100%	ABIIQX030000005.1
419	*Acinetobacter baumannii*	Acinetobacter baumannii strain 2022HL-01688 SAMN30889194-rid20906983.denovo.08	100%	100%	ABIIRD030000008.1
420	*Acinetobacter baumannii*	Acinetobacter baumannii strain 2022HL-01684 SAMN30889190-rid20914733.denovo.08	100%	100%	ABIHOM030000008.1
421	*Acinetobacter baumannii*	Acinetobacter baumannii strain 2022HL-01683 SAMN30889189-rid20917193.denovo.11	100%	100%	ABIHQW030000011.1
422	*Acinetobacter baumannii*	Acinetobacter baumannii strain 2022HL-01657 SAMN30498068-rid20852403.denovo.11	100%	100%	ABHTPT030000011.1
423	*Acinetobacter baumannii*	Acinetobacter baumannii strain 2022HL-01654 SAMN30498065-rid20881533.denovo.03	100%	100%	ABHTPQ030000003.1
424	*Acinetobacter baumannii*	Acinetobacter baumannii strain 2022HL-01651 SAMN30498062-rid20881543.denovo.09	100%	100%	ABHTPN030000009.1
425	*Acinetobacter baumannii*	Acinetobacter baumannii strain 2022HL-01494 SAMN29869686-rid20864543.denovo.10	100%	100%	ABHFFQ030000010.1
426	*Acinetobacter baumannii*	Acinetobacter baumannii strain 2022HL-01493 SAMN29869685-rid20877863.denovo.09	100%	100%	ABHFGS030000009.1
427	*Acinetobacter baumannii*	Acinetobacter baumannii strain 2022HL-01475 SAMN29869133-rid20877843.denovo.11	100%	100%	ABHFDR030000011.1
428	*Acinetobacter baumannii*	Acinetobacter baumannii strain 2022HL-01441 SAMN29507305-rid20848903.denovo.11	100%	100%	ABGYJA030000011.1
429	*Acinetobacter baumannii*	Acinetobacter baumannii strain 2022HL-01399 SAMN29507156-rid20894113.denovo.05	100%	100%	ABGYLC030000005.1
430	*Acinetobacter baumannii*	Acinetobacter baumannii strain 2022HL-01394 SAMN29507151-rid20863443.denovo.10	100%	100%	ABGYAU030000010.1
431	*Acinetobacter baumannii*	Acinetobacter baumannii strain 2022HL-01269 SAMN28194893-rid20899003.denovo.11	100%	100%	ABGDYE030000011.1
432	*Acinetobacter baumannii*	Acinetobacter baumannii strain 2022HL-01193 SAMN27480464-rid20875623.denovo.03	100%	100%	ABFOGX030000003.1
433	*Acinetobacter baumannii*	Acinetobacter baumannii strain 2021HL-00893 SAMN26365926-rid20884273.denovo.08	100%	100%	ABFGUE030000008.1
434	*Acinetobacter baumannii*	Acinetobacter baumannii strain 2021HL-00889 SAMN26365844-rid20893933.denovo.09	100%	100%	ABFGTJ030000009.1
435	*Acinetobacter baumannii*	Acinetobacter baumannii strain 2021HL-00887 SAMN26365842-rid20901163.denovo.06	100%	100%	ABFGTH030000006.1
436	*Acinetobacter baumannii*	Acinetobacter baumannii strain 2021HL-00883 SAMN26365838-rid20875563.denovo.19	100%	100%	ABFGSQ030000017.1
437	*Acinetobacter baumannii*	Acinetobacter baumannii strain 2021HL-00871 SAMN26365826-rid20850663.denovo.10	100%	100%	ABFGSU030000010.1
438	*Acinetobacter baumannii*	Acinetobacter baumannii strain 2021GO-0199 SAMN23521417-rid20949213.denovo.06	100%	100%	ABECBX030000006.1
439	*Acinetobacter baumannii*	Acinetobacter baumannii strain 2021GO-0192 SAMN23521410-rid20929103.denovo.07	100%	100%	ABECBQ030000007.1
440	*Acinetobacter baumannii*	Acinetobacter baumannii strain 2021GO-0159 SAMN22442194-rid20914133.denovo.053	100%	100%	ABDIYM030000053.1
441	*Acinetobacter baumannii*	Acinetobacter baumannii strain 2021GO-0129 SAMN22045169-rid20892643.denovo.041	100%	100%	ABCZEH030000040.1
442	*Acinetobacter baumannii*	Acinetobacter baumannii strain 2021GO-0115 SAMN22045155-rid20920303.denovo.073	100%	100%	ABCXDG030000073.1
443	*Acinetobacter baumannii*	Acinetobacter baumannii strain 2021GO-0112 SAMN22442183-rid20875403.denovo.045	100%	100%	ABDJBJ030000042.1
444	*Acinetobacter baumannii*	Acinetobacter baumannii strain 2021GO-0106 SAMN22045147-rid20916343.denovo.175	100%	100%	ABCXED030000169.1
445	*Acinetobacter baumannii*	Acinetobacter baumannii strain 2021GO-0104 SAMN22045145-rid20921203.denovo.063	100%	100%	ABCZEJ030000063.1
446	*Acinetobacter baumannii*	Acinetobacter baumannii strain 2021GO-0103 SAMN22045144-rid20915373.denovo.043	100%	100%	ABCXDV030000042.1
447	*Acinetobacter baumannii*	Acinetobacter baumannii strain 2021GO-0071 SAMN19715144-rid20896583.denovo.068	100%	100%	ABAXEK030000067.1
448	*Acinetobacter baumannii*	Acinetobacter baumannii strain 2021GO-0055 SAMN19715129-rid20919363.denovo.04	100%	100%	ABAXEQ030000004.1
449	*Acinetobacter baumannii*	Acinetobacter baumannii strain 2021GO-0054 SAMN19715128-rid20927843.denovo.05	100%	100%	ABAXEO030000005.1
450	*Acinetobacter baumannii*	Acinetobacter baumannii strain 2021GO-0036 SAMN19022529-rid20851973.denovo.072	100%	100%	ABAKCK030000070.1
451	*Acinetobacter baumannii*	Acinetobacter baumannii strain 2021GO-0035 SAMN19022528-rid20867233.denovo.040	100%	100%	ABAKCL030000040.1
452	*Acinetobacter baumannii*	Acinetobacter baumannii strain 2021GO-0034 SAMN19022527-rid20866543.denovo.073	100%	100%	ABAKCM030000072.1
453	*Acinetobacter baumannii*	Acinetobacter baumannii strain 2021GO-0029 SAMN19022522-rid20950413.denovo.04	100%	100%	ABAKCI030000004.1
454	*Acinetobacter baumannii*	Acinetobacter baumannii strain 2021GO-0009 SAMN19022502-rid20875163.denovo.043	100%	100%	ABAKBH030000043.1
455	*Acinetobacter baumannii*	Acinetobacter baumannii strain 2021GO-0006 SAMN19022499-rid20869503.denovo.066	100%	100%	ABAKBM030000063.1
456	*Acinetobacter baumannii*	Acinetobacter baumannii strain 2021GO-0005 SAMN19022498-rid20875173.denovo.015	100%	100%	ABAKBR030000015.1
457	*Acinetobacter baumannii*	Acinetobacter baumannii strain 2020HL-00752 NODE_35_length_21605_cov_59.633206	100%	100%	AAYLMM040000035.1
458	*Acinetobacter baumannii*	Acinetobacter baumannii strain 2020HL-00708 SAMN16940762-rid20898603.denovo.10	100%	100%	AAYLNG030000010.1
459	*Acinetobacter baumannii*	Acinetobacter baumannii strain 2020HL-00706 SAMN16940690-rid20908173.denovo.12	100%	100%	AAYLOD030000012.1
460	*Acinetobacter baumannii*	Acinetobacter baumannii strain 2020HL-00702 SAMN16940686-rid20844743.denovo.056	100%	100%	AAYLNH030000052.1
461	*Acinetobacter baumannii*	Acinetobacter baumannii strain 2020HL-00701 SAMN16940685-rid20871283.denovo.09	100%	100%	AAYLNL030000009.1
462	*Acinetobacter baumannii*	Acinetobacter baumannii strain 2020HL-00697 SAMN16940681-rid20867143.denovo.10	100%	100%	AAYLNS030000010.1
463	*Acinetobacter baumannii*	Acinetobacter baumannii strain 2020HL-00490 SAMN16829380-rid20883923.denovo.09	100%	100%	AAYRRN030000009.1
464	*Acinetobacter baumannii*	Acinetobacter baumannii strain 2020HL-00489 SAMN16829379-rid20867113.denovo.08	100%	100%	AAYLQZ030000008.1
465	*Acinetobacter baumannii*	Acinetobacter baumannii strain 2020HL-00488 SAMN16829378-rid20872083.denovo.10	100%	100%	AAYLQP030000010.1
466	*Acinetobacter baumannii*	Acinetobacter baumannii strain 2020HL-00484 SAMN16829374-rid26886453.denovo.10	100%	100%	AAYLQC040000010.1

467	*Acinetobacter baumannii*	Acinetobacter baumannii strain 2020HL-00483 SAMN16829373-rid20938063.denovo.11	100%	100%	AAYRRK030000011.1
468	*Acinetobacter baumannii*	Acinetobacter baumannii strain 2020HL-00482 SAMN16829372-rid26885763.denovo.11	100%	100%	AAYLQT040000011.1
469	*Acinetobacter baumannii*	Acinetobacter baumannii strain 2020HL-00480 SAMN16829370-rid20898573.denovo.09	100%	100%	AAYLQS030000009.1
470	*Acinetobacter baumannii*	Acinetobacter baumannii strain 2020HL-00479 SAMN16829369-rid20906053.denovo.10	100%	100%	AAYLQQ030000010.1
471	*Acinetobacter baumannii*	Acinetobacter baumannii strain 2020HL-00478 SAMN16829368-rid20906063.denovo.10	100%	100%	AAYLQR030000010.1
472	*Acinetobacter baumannii*	Acinetobacter baumannii strain 2020HL-00477 SAMN16829367-rid20912743.denovo.01	100%	100%	AAYLQX030000001.1
473	*Acinetobacter baumannii*	Acinetobacter baumannii strain 2020HL-00474 SAMN16829364-rid20874973.denovo.01	100%	100%	AAYLQY030000001.1
474	*Acinetobacter baumannii*	Acinetobacter baumannii strain 2020HL-00473 SAMN16829363-rid20882723.denovo.01	100%	100%	AAYLQG030000001.1
475	*Acinetobacter baumannii*	Acinetobacter baumannii strain 2020HL-00472 SAMN16829362-rid20893533.denovo.10	100%	100%	AAYLQE030000010.1
476	*Acinetobacter baumannii*	Acinetobacter baumannii strain 2020HL-00471 SAMN16829361-rid20898583.denovo.09	100%	100%	AAYLQF030000009.1
477	*Acinetobacter baumannii*	Acinetobacter baumannii strain 2020HL-00465 SAMN16829355-rid20906043.denovo.10	100%	100%	AAYRRP030000010.1
478	*Acinetobacter baumannii*	Acinetobacter baumannii strain 2020HL-00429 SAMN16829417-rid20939273.denovo.10	100%	100%	AAYLQM030000010.1
479	*Acinetobacter baumannii*	Acinetobacter baumannii strain 2020HL-00418 SAMN16878211-rid20920263.denovo.10	100%	100%	AAYRRI030000010.1
480	*Acinetobacter baumannii*	Acinetobacter baumannii strain 2020HL-00417 SAMN16878210-rid20892463.denovo.12	100%	100%	AAYLRB030000012.1
481	*Acinetobacter baumannii*	Acinetobacter baumannii strain 2020HL-00415 SAMN16878208-rid20903443.denovo.03	100%	100%	AAYLQD030000003.1
482	*Acinetobacter baumannii*	Acinetobacter baumannii strain 2020HL-00413 SAMN16878206-rid20892473.denovo.10	100%	100%	AAYLQJ030000010.1
483	*Acinetobacter baumannii*	Acinetobacter baumannii strain 2020GO-0197 SAMN19022487-rid20848593.denovo.024	100%	100%	ABAKBG030000023.1
484	*Acinetobacter baumannii*	Acinetobacter baumannii strain 2020GO-0193 SAMN19022483-rid20844823.denovo.076	100%	100%	ABAKDC030000074.1
485	*Acinetobacter baumannii*	Acinetobacter baumannii strain 2020GO-00183 SAMN17767364-rid20877163.denovo.029	100%	100%	AAZDXA030000029.1
486	*Acinetobacter baumannii*	Acinetobacter baumannii strain 2020GO-00177 SAMN17767358-rid20848123.denovo.067	100%	100%	AAZDWJ030000065.1
487	*Acinetobacter baumannii*	Acinetobacter baumannii strain 2020GO-00162 SAMN17767343-rid20913823.denovo.067	100%	100%	AAZDWC030000064.1
488	*Acinetobacter baumannii*	Acinetobacter baumannii strain 2020GO-00161 SAMN17767342-rid20898653.denovo.078	100%	100%	AAZDWB030000076.1
489	*Acinetobacter baumannii*	Acinetobacter baumannii strain 2020GO-00155 SAMN17767336-rid20845883.denovo.017	100%	100%	AAZDVP030000016.1
490	*Acinetobacter baumannii*	Acinetobacter baumannii strain 2020GO-00135 SAMN17767316-rid20941353.denovo.048	100%	100%	AAZDVO030000046.1
491	*Acinetobacter baumannii*	Acinetobacter baumannii strain 2020GO-00131 SAMN17767312-rid20930223.denovo.050	100%	100%	AAZDUX030000050.1
492	*Acinetobacter baumannii*	Acinetobacter baumannii strain 2020GO-00120 SAMN17767301-rid20869463.denovo.031	100%	100%	AAZDUS030000031.1
493	*Acinetobacter baumannii*	Acinetobacter baumannii strain 2020GO-00119 SAMN17767300-rid20873013.denovo.035	100%	100%	AAZDUT030000034.1
494	*Acinetobacter baumannii*	Acinetobacter baumannii strain 2020GO-00110 SAMN17767291-rid20875083.denovo.037	100%	100%	AAZDUO030000035.1
495	*Acinetobacter baumannii*	Acinetobacter baumannii strain 2020GO-00096 SAMN17767277-rid20893563.denovo.35	100%	100%	AAZDVR030000034.1
496	*Acinetobacter baumannii*	Acinetobacter baumannii strain 2020GO-00079 SAMN16552563-rid20894743.denovo.028	100%	100%	AAYLUM030000026.1
497	*Acinetobacter baumannii*	Acinetobacter baumannii strain 2020GO-00060 SAMN16552544-rid20896463.denovo.042	100%	100%	AAYLUL030000041.1
498	*Acinetobacter baumannii*	Acinetobacter baumannii strain 2020GO-00029 SAMN16552513-rid20874913.denovo.051	100%	100%	AAYRRT030000048.1
499	*Acinetobacter baumannii*	Acinetobacter baumannii strain 2019HL-00621 SAMN16940002-rid20890373.denovo.070	100%	100%	AAYLOE030000068.1
500	*Acinetobacter baumannii*	Acinetobacter baumannii strain 2019HL-00326 SAMN16842685-rid20904623.denovo.073	100%	100%	AAYRRS030000069.1
501	*Acinetobacter baumannii*	Acinetobacter baumannii strain 2019GO-00102 SAMN14669292-rid20877043.denovo.068	100%	100%	AAYMFM030000065.1
502	*Acinetobacter baumannii*	Acinetobacter baumannii strain 2019GO-00099 SAMN14669289-rid20872893.denovo.015	100%	100%	AAYMHO030000015.1
503	*Acinetobacter baumannii*	Acinetobacter baumannii strain 2019GO-00059 SAMN14669249-rid20913693.denovo.080	100%	100%	AAYMGT030000078.1
504	*Acinetobacter baumannii*	Acinetobacter baumannii strain 2019GO-00018 SAMN14669208-rid20915973.denovo.14	100%	100%	AAYMGB030000014.1
505	*Acinetobacter baumannii*	Acinetobacter baumannii strain 2018HL-01509 SAMN30349737-rid20869983.denovo.073	100%	100%	ABHYHZ030000070.1
506	*Acinetobacter baumannii*	Acinetobacter baumannii strain 2018HL-01506 1	100%	100%	ABHYIO040000001.1
507	*Acinetobacter baumannii*	Acinetobacter baumannii strain 2018HL-01504 SAMN30349732-rid20898033.denovo.078	100%	100%	ABHYIM030000076.1
508	*Acinetobacter baumannii*	Acinetobacter baumannii strain 2018HL-01502 SAMN30349730-rid20877913.denovo.062	100%	100%	ABHYIK030000060.1
509	*Acinetobacter baumannii*	Acinetobacter baumannii strain 2018HL-01498 SAMN30349726-rid20930313.denovo.084	100%	100%	ABHYHX030000084.1
510	*Acinetobacter baumannii*	Acinetobacter baumannii strain 2018HL-00608 SAMN16939989-rid20872093.denovo.075	100%	100%	AAYLOX030000074.1
511	*Acinetobacter baumannii*	Acinetobacter baumannii strain 2018HL-00607 SAMN16939988-rid20893543.denovo.069	100%	100%	AAYRRD030000069.1
512	*Acinetobacter baumannii*	Acinetobacter baumannii strain 2018HL-00603 SAMN16939984-rid20882733.denovo.082	100%	100%	AAYLOR030000080.1
513	*Acinetobacter baumannii*	Acinetobacter baumannii strain 2018HL-00592 SAMN16939973-rid20853063.denovo.064	100%	100%	AAYLOO030000062.1
514	*Acinetobacter baumannii*	Acinetobacter baumannii strain 2018HL-00591 SAMN16939972-rid20903463.denovo.066	100%	100%	AAYLOI030000065.1
515	*Acinetobacter baumannii*	Acinetobacter baumannii strain 2018HL-00579 SAMN16939959-rid20897543.denovo.087	100%	100%	AAYLOQ030000084.1
516	*Acinetobacter baumannii*	Acinetobacter baumannii strain 2018HL-00571 SAMN16939951-rid20865303.denovo.091	100%	100%	AAYLPC030000087.1
517	*Acinetobacter baumannii*	Acinetobacter baumannii strain 2018HL-00569 SAMN16939949-rid20903453.denovo.088	100%	100%	AAYLPF030000086.1
518	*Acinetobacter baumannii*	Acinetobacter baumannii strain 2018HL-00515 SAMN16916280-rid20870703.denovo.073	100%	100%	AAYLPS030000070.1
519	*Acinetobacter baumannii*	Acinetobacter baumannii strain 2018HL-00511 SAMN16916276-rid20919303.denovo.074	100%	100%	AAYLPQ030000074.1
520	*Acinetobacter baumannii*	Acinetobacter baumannii strain 2018HL-00510 SAMN16916275-rid20928983.denovo.085	100%	100%	AAYLPT030000085.1
521	*Acinetobacter baumannii*	Acinetobacter baumannii strain 2018HL-00259 SAMN16895803-rid20900403.denovo.077	100%	100%	AAYLPM030000075.1
522	*Acinetobacter baumannii*	Acinetobacter baumannii strain 2018HL-00240 SAMN16895784-rid20863023.denovo.038	100%	100%	AAYRRL030000036.1
523	*Acinetobacter baumannii*	Acinetobacter baumannii strain 2018HL-00238 SAMN16895782-rid20874993.denovo.075	100%	100%	AAYLPY030000073.1
524	*Acinetobacter baumannii*	Acinetobacter baumannii strain 2018HL-00220 SAMN16895742-rid20872963.denovo.071	100%	100%	AAYLQA030000067.1
525	*Acinetobacter baumannii*	Acinetobacter baumannii strain 2018HL-00219 SAMN16895741-rid20900393.denovo.074	100%	100%	AAYLPZ030000070.1
526	*Acinetobacter baumannii*	Acinetobacter baumannii strain 2018HL-00063 SAMN16823382-rid20877103.denovo.046	100%	100%	AAYLSB030000046.1
527	*Acinetobacter baumannii*	Acinetobacter baumannii strain 2018HL-00062 SAMN16823381-rid20897523.denovo.075	100%	100%	AAYLSG030000073.1
528	*Acinetobacter baumannii*	Acinetobacter baumannii strain 2018HL-00061 SAMN16823380-rid20872943.denovo.061	100%	100%	AAYLSC030000059.1
529	*Acinetobacter baumannii*	Acinetobacter baumannii strain 2018HL-00059 SAMN16823378-rid20917923.denovo.070	100%	100%	AAYLSF030000070.1
530	*Acinetobacter baumannii*	Acinetobacter baumannii strain 2017HL-01501 SAMN30349729-rid20927983.denovo.074	100%	100%	ABHYIJ030000074.1
531	*Acinetobacter baumannii*	Acinetobacter baumannii strain 2017HL-01500 SAMN30349728-rid20917163.denovo.070	100%	100%	ABHYII030000070.1
532	*Acinetobacter baumannii*	Acinetobacter baumannii strain 17A1955 ctg_100	100%	100%	JAIWUA010000100.1
533	*Acinetobacter baumannii*	Acinetobacter baumannii strain 172315 NODE_17_length_19594_cov_116.561348	100%	100%	JABJRM010000017.1
534	*Acinetobacter baumannii*	Acinetobacter baumannii strain 149-AB SAMD00555928-rid21359403.denovo.156	100%	100%	DAXUSQ010000146.1
535	*Acinetobacter baumannii*	Acinetobacter baumannii strain 149_59283_AB_2014 SAMD00330550-rid26698873.denovo.156	100%	100%	DBJDPO010000146.1
536	*Acinetobacter baumannii*	Acinetobacter baumannii strain 13ARS_MAR0194 SAMEA25885918-rid6942183.denovo.30	100%	100%	DADBIE010000028.1
537	*Acinetobacter baumannii*	Acinetobacter baumannii strain 13ARS_CVM0021 SAMEA25870168-rid6941973.denovo.36	100%	100%	DADBIT010000033.1
538	*Acinetobacter baumannii*	Acinetobacter baumannii isolate whole organism SAMN33193788-rid20909033.denovo.12	100%	100%	ABKVAC030000012.1
539	*Acinetobacter baumannii*	Acinetobacter baumannii isolate whole organism SAMN33193787-rid20926943.denovo.12	100%	100%	ABKVAB030000012.1
540	*Acinetobacter baumannii*	Acinetobacter baumannii isolate whole organism SAMN33193786-rid20907303.denovo.12	100%	100%	ABKVAA030000012.1
541	*Acinetobacter baumannii*	Acinetobacter baumannii isolate human SAMN47990436-rid26031003.denovo.10	100%	100%	ABYQWN010000010.1
542	*Acinetobacter baumannii*	Acinetobacter baumannii isolate human SAMN42581481-rid22364293.denovo.09	100%	100%	ABTNZP010000009.1
543	*Acinetobacter baumannii*	Acinetobacter baumannii isolate BAL 255 genome assembly, contig: scaffold59	100%	100%	CZWB01000059.1
544	*Acinetobacter baumannii*	Acinetobacter baumannii isolate Acinetobacter baumannii SAMEA112328331-rid16981523.denovo.20	100%	100%	DAOHRM010000020.1
545	*Acinetobacter baumannii*	Acinetobacter baumannii isolate Acinetobacter baumannii SAMEA112328323-rid16981503.denovo.016	100%	100%	DANEQT010000016.1
546	*Acinetobacter baumannii*	Acinetobacter baumannii isolate Acinetobacter baumannii SAMEA112328322-rid16816693.denovo.10	100%	100%	DAMXNB010000010.1
547	*Acinetobacter baumannii*	Acinetobacter baumannii isolate Acinetobacter baumannii SAMEA112328307-rid16870913.denovo.012	100%	100%	DAMZCZ010000012.1
548	*Acinetobacter baumannii*	Acinetobacter baumannii isolate Acinetobacter baumannii SAMEA112328300-rid17065013.denovo.078	100%	100%	DANIML010000078.1
549	*Acinetobacter baumannii*	Acinetobacter baumannii isolate 2025DU-00149 SAMN48710319-rid26515513.denovo.10	100%	100%	ABZCJT010000010.1
550	*Acinetobacter baumannii*	Acinetobacter baumannii isolate 2025DU-00099 SAMN47210778-rid25540293.denovo.05	100%	100%	ABYKPC010000005.1
551	*Acinetobacter baumannii*	Acinetobacter baumannii isolate 2025DU-00098 SAMN47210779-rid25540303.denovo.11	100%	100%	ABYKPB010000011.1
552	*Acinetobacter baumannii*	Acinetobacter baumannii isolate 2025DU-00097 SAMN47210780-rid25540313.denovo.12	100%	100%	ABYKOZ010000012.1
553	*Acinetobacter baumannii*	Acinetobacter baumannii isolate 2025DU-00093 SAMN47210784-rid25540353.denovo.11	100%	100%	ABYKOV010000011.1
554	*Acinetobacter baumannii*	Acinetobacter baumannii isolate 2025DU-00091 SAMN47210786-rid25540373.denovo.13	100%	100%	ABYKOW010000013.1
555	*Acinetobacter baumannii*	Acinetobacter baumannii isolate 2025DU-00090 SAMN47210787-rid25540383.denovo.52	100%	100%	ABYKOT010000046.1
556	*Acinetobacter baumannii*	Acinetobacter baumannii isolate 2025DU-00088 SAMN46987791-rid25439043.denovo.12	100%	100%	ABYABM010000012.1
557	*Acinetobacter baumannii*	Acinetobacter baumannii isolate 2025DU-00087 SAMN46987792-rid25439053.denovo.11	100%	100%	ABYABL010000011.1
558	*Acinetobacter baumannii*	Acinetobacter baumannii isolate 2025DU-00085 SAMN46987794-rid25439073.denovo.10	100%	100%	ABYABF010000010.1
559	*Acinetobacter baumannii*	Acinetobacter baumannii isolate 2025DU-00083 SAMN46987796-rid25439093.denovo.12	100%	100%	ABYABC010000012.1
560	*Acinetobacter baumannii*	Acinetobacter baumannii isolate 2025DU-00078 SAMN46987801-rid25439143.denovo.12	100%	100%	ABYAFD010000012.1
561	*Acinetobacter baumannii*	Acinetobacter baumannii isolate 2025DU-00077 SAMN46987802-rid25439153.denovo.10	100%	100%	ABYAAX010000010.1
562	*Acinetobacter baumannii*	Acinetobacter baumannii isolate 2025DU-00073 SAMN46880491-rid25338443.denovo.12	100%	100%	ABXXWC010000012.1
563	*Acinetobacter baumannii*	Acinetobacter baumannii isolate 2025DU-00071 SAMN46880493-rid25338463.denovo.11	100%	100%	ABXXYH010000011.1
564	*Acinetobacter baumannii*	Acinetobacter baumannii isolate 2025DU-00070 SAMN46880494-rid25338473.denovo.11	100%	100%	ABXXQO010000011.1
565	*Acinetobacter baumannii*	Acinetobacter baumannii isolate 2025DU-00068 SAMN46880496-rid25338493.denovo.11	100%	100%	ABXXVY010000011.1
566	*Acinetobacter baumannii*	Acinetobacter baumannii isolate 2025DU-00066 SAMN46880498-rid25338513.denovo.12	100%	100%	ABXXQM010000012.1
567	*Acinetobacter baumannii*	Acinetobacter baumannii isolate 2025DU-00065 SAMN46880499-rid25338523.denovo.11	100%	100%	ABXXYG010000011.1
568	*Acinetobacter baumannii*	Acinetobacter baumannii isolate 2025DU-00064 SAMN46880500-rid25338533.denovo.05	100%	100%	ABXXVZ010000005.1
569	*Acinetobacter baumannii*	Acinetobacter baumannii isolate 2025DU-00063 SAMN46880501-rid25338543.denovo.10	100%	100%	ABXXQL010000010.1
570	*Acinetobacter baumannii*	Acinetobacter baumannii isolate 2025DU-00061 SAMN46819733-rid25326263.denovo.11	100%	100%	ABXUQF010000011.1
571	*Acinetobacter baumannii*	Acinetobacter baumannii isolate 2025DU-00059 SAMN46819735-rid25326283.denovo.12	100%	100%	ABXUQB010000012.1
572	*Acinetobacter baumannii*	Acinetobacter baumannii isolate 2025DU-00050 SAMN46759048-rid25288623.denovo.09	100%	100%	ABXRME010000009.1
573	*Acinetobacter baumannii*	Acinetobacter baumannii isolate 2025DU-00048 SAMN46759050-rid25288643.denovo.09	100%	100%	ABXRMC010000009.1
574	*Acinetobacter baumannii*	Acinetobacter baumannii isolate 2025DU-00047 SAMN46759051-rid25288653.denovo.09	100%	100%	ABXRMB010000009.1
575	*Acinetobacter baumannii*	Acinetobacter baumannii isolate 2025DU-00045 SAMN46712523-rid25157753.denovo.10	100%	100%	ABXNMA010000010.1
576	*Acinetobacter baumannii*	Acinetobacter baumannii isolate 2025DU-00042 SAMN46439165-rid24971913.denovo.10	100%	100%	ABXGTM010000010.1
577	*Acinetobacter baumannii*	Acinetobacter baumannii isolate 2025DU-00040 SAMN46439167-rid24971933.denovo.12	100%	100%	ABXGTK010000012.1
578	*Acinetobacter baumannii*	Acinetobacter baumannii isolate 2025DU-00039 SAMN46439168-rid24971943.denovo.12	100%	100%	ABXGSS010000012.1
579	*Acinetobacter baumannii*	Acinetobacter baumannii isolate 2025DU-00037 SAMN46439170-rid24971963.denovo.10	100%	100%	ABXGSP010000010.1
580	*Acinetobacter baumannii*	Acinetobacter baumannii isolate 2025DU-00035 SAMN46439172-rid24971983.denovo.11	100%	100%	ABXGTI010000011.1
581	*Acinetobacter baumannii*	Acinetobacter baumannii isolate 2025DU-00034 SAMN46439173-rid24971993.denovo.12	100%	100%	ABXGTG010000012.1
582	*Acinetobacter baumannii*	Acinetobacter baumannii isolate 2025DU-00032 SAMN46439175-rid24972013.denovo.11	100%	100%	ABXGSO010000011.1
583	*Acinetobacter baumannii*	Acinetobacter baumannii isolate 2025DU-00031 SAMN46439176-rid24972023.denovo.12	100%	100%	ABXGSM010000012.1

584	*Acinetobacter baumannii*	Acinetobacter baumannii isolate 2025DU-00030 SAMN46439177-rid24972033.denovo.13	100%	100%	ABXGSL010000013.1
585	*Acinetobacter baumannii*	Acinetobacter baumannii isolate 2025DU-00029 SAMN46439178-rid24972043.denovo.12	100%	100%	ABXGSK010000012.1
586	*Acinetobacter baumannii*	Acinetobacter baumannii isolate 2025DU-00027 SAMN46439180-rid24972063.denovo.09	100%	100%	ABXGTF010000009.1
587	*Acinetobacter baumannii*	Acinetobacter baumannii isolate 2025DU-00024 SAMN46439183-rid24972093.denovo.09	100%	100%	ABXGSN010000009.1
588	*Acinetobacter baumannii*	Acinetobacter baumannii isolate 2025DU-00022 SAMN46401068-rid24970903.denovo.11	100%	100%	ABXGSX010000011.1
589	*Acinetobacter baumannii*	Acinetobacter baumannii isolate 2025DU-00020 SAMN46401070-rid24970923.denovo.10	100%	100%	ABXGSZ010000010.1
590	*Acinetobacter baumannii*	Acinetobacter baumannii isolate 2025DU-00019 SAMN46401071-rid24970933.denovo.12	100%	100%	ABXVXU010000012.1
591	*Acinetobacter baumannii*	Acinetobacter baumannii isolate 2025DU-00017 SAMN46401073-rid24970953.denovo.16	100%	100%	ABXGSU010000016.1
592	*Acinetobacter baumannii*	Acinetobacter baumannii isolate 2025DU-00014 SAMN46401076-rid24970983.denovo.10	100%	100%	ABXGTH010000010.1
593	*Acinetobacter baumannii*	Acinetobacter baumannii isolate 2025DU-00013 SAMN46401077-rid24970993.denovo.10	100%	100%	ABXGSQ010000010.1
594	*Acinetobacter baumannii*	Acinetobacter baumannii isolate 2025DU-00011 SAMN46293075-rid24921323.denovo.12	100%	100%	ABXDNE010000012.1
595	*Acinetobacter baumannii*	Acinetobacter baumannii isolate 2025DU-00007 SAMN46293079-rid24921363.denovo.43	100%	100%	ABXDMP010000037.1
596	*Acinetobacter baumannii*	Acinetobacter baumannii isolate 2025DU-00004 SAMN46293082-rid24921393.denovo.12	100%	100%	ABXDMM010000012.1
597	*Acinetobacter baumannii*	Acinetobacter baumannii isolate 2025DU-00002 SAMN46293084-rid24921413.denovo.12	100%	100%	ABXDMK010000012.1
598	*Acinetobacter baumannii*	Acinetobacter baumannii isolate 2025DU-00001 SAMN46293085-rid24921423.denovo.12	100%	100%	ABXDMJ010000012.1
599	*Acinetobacter baumannii*	Acinetobacter baumannii isolate 2025CB-00211 SAMN47512745-rid25737243.denovo.51	100%	100%	ABYHUC010000046.1
600	*Acinetobacter baumannii*	Acinetobacter baumannii isolate 2025BM-00086 SAMN50086147-rid26942613.denovo.10	100%	100%	ABZTCY010000010.1
601	*Acinetobacter baumannii*	Acinetobacter baumannii isolate 2025BM-00085 SAMN50086146-rid26943583.denovo.10	100%	100%	ABZTCX010000010.1
602	*Acinetobacter baumannii*	Acinetobacter baumannii isolate 2025BM-00082 SAMN49995114-rid26913003.denovo.12	100%	100%	ABZSKE010000012.1
603	*Acinetobacter baumannii*	Acinetobacter baumannii isolate 2025BM-00071 SAMN49096994-rid26704603.denovo.11	100%	100%	ABZJFI010000011.1
604	*Acinetobacter baumannii*	Acinetobacter baumannii isolate 2025BM-00070 SAMN49018426-rid26681023.denovo.11	100%	100%	ABZIAX010000011.1
605	*Acinetobacter baumannii*	Acinetobacter baumannii isolate 2025BM-00069 SAMN49018425-rid26681033.denovo.12	100%	100%	ABZIAY010000012.1
606	*Acinetobacter baumannii*	Acinetobacter baumannii isolate 2025BM-00065 SAMN48984433-rid26668083.denovo.09	100%	100%	ABZHSH010000009.1
607	*Acinetobacter baumannii*	Acinetobacter baumannii isolate 2025BM-00059 SAMN48523854-rid26431673.denovo.11	100%	100%	ABYZEI010000011.1
608	*Acinetobacter baumannii*	Acinetobacter baumannii isolate 2025BM-00057 SAMN48346123-rid26344483.denovo.11	100%	100%	ABYWBI010000011.1
609	*Acinetobacter baumannii*	Acinetobacter baumannii isolate 2025BM-00056 SAMN48346122-rid26344493.denovo.11	100%	100%	ABYWCF010000011.1
610	*Acinetobacter baumannii*	Acinetobacter baumannii isolate 2025BM-00050 SAMN48178201-rid26203343.denovo.10	100%	100%	ABYULQ010000010.1
611	*Acinetobacter baumannii*	Acinetobacter baumannii isolate 2025BM-00031 SAMN47290616-rid25617543.denovo.12	100%	100%	ABYIUJ010000012.1
612	*Acinetobacter baumannii*	Acinetobacter baumannii isolate 2025BM-00027 SAMN47226395-rid25558103.denovo.12	100%	100%	ABYKIS010000012.1
613	*Acinetobacter baumannii*	Acinetobacter baumannii isolate 2025BM-00013 SAMN46527816-rid25053753.denovo.13	100%	100%	ABXLJE010000013.1
614	*Acinetobacter baumannii*	Acinetobacter baumannii isolate 2025BM-00004 SAMN46231628-rid24710663.denovo.12	100%	100%	ABXAAR010000012.1
615	*Acinetobacter baumannii*	Acinetobacter baumannii isolate 2024EH-00039 SAMN44245481-rid23616323.denovo.001	100%	100%	ABVVXJ010000001.1
616	*Acinetobacter baumannii*	Acinetobacter baumannii isolate 2024DU00076 SAMN45989680-rid24627613.denovo.09	100%	100%	ABWXWA010000009.1
617	*Acinetobacter baumannii*	Acinetobacter baumannii isolate 2024DU00075 SAMN45989681-rid24627763.denovo.12	100%	100%	ABWXVZ010000012.1
618	*Acinetobacter baumannii*	Acinetobacter baumannii isolate 2024DU00074 SAMN45989682-rid24627773.denovo.10	100%	100%	ABWXVY010000010.1
619	*Acinetobacter baumannii*	Acinetobacter baumannii isolate 2024DU00072 SAMN45926131-rid24585533.denovo.12	100%	100%	ABWXAU010000012.1
620	*Acinetobacter baumannii*	Acinetobacter baumannii isolate 2024DU00069 SAMN45926134-rid24585563.denovo.12	100%	100%	ABWXAP010000012.1
621	*Acinetobacter baumannii*	Acinetobacter baumannii isolate 2024DU00067 SAMN45895297-rid24572383.denovo.11	100%	100%	ABWWSO010000011.1
622	*Acinetobacter baumannii*	Acinetobacter baumannii isolate 2024DU00066 SAMN45895295-rid24572393.denovo.10	100%	100%	ABWWSC010000010.1
623	*Acinetobacter baumannii*	Acinetobacter baumannii isolate 2024DU00064 SAMN45895294-rid24572413.denovo.10	100%	100%	ABWWSB010000010.1
624	*Acinetobacter baumannii*	Acinetobacter baumannii isolate 2024DU00061 SAMN45895299-rid24572443.denovo.12	100%	100%	ABWWRY010000012.1
625	*Acinetobacter baumannii*	Acinetobacter baumannii isolate 2024DU00058 SAMN45895304-rid24572473.denovo.11	100%	100%	ABWWRT010000011.1
626	*Acinetobacter baumannii*	Acinetobacter baumannii isolate 2024DU00057 SAMN45895303-rid24572483.denovo.12	100%	100%	ABWWRS010000012.1
627	*Acinetobacter baumannii*	Acinetobacter baumannii isolate 2024DU00055 SAMN45113782-rid24258153.denovo.12	100%	100%	ABWNFK010000012.1
628	*Acinetobacter baumannii*	Acinetobacter baumannii isolate 2024DU00052 SAMN45113783-rid24258183.denovo.10	100%	100%	ABWNFI010000010.1
629	*Acinetobacter baumannii*	Acinetobacter baumannii isolate 2024DU00050 SAMN45113780-rid24258203.denovo.12	100%	100%	ABWNFF010000012.1
630	*Acinetobacter baumannii*	Acinetobacter baumannii isolate 2024DU00046 SAMN45113778-rid24258233.denovo.11	100%	100%	ABWNFA010000011.1
631	*Acinetobacter baumannii*	Acinetobacter baumannii isolate 2024DU00044 SAMN44842728-rid24048283.denovo.12	100%	100%	ABWLHX010000012.1
632	*Acinetobacter baumannii*	Acinetobacter baumannii isolate 2024DU00041 SAMN44842732-rid24052063.denovo.11	100%	100%	ABWLPA010000011.1
633	*Acinetobacter baumannii*	Acinetobacter baumannii isolate 2024DU00033 SAMN44842722-rid24048313.denovo.12	100%	100%	ABWLHR010000012.1
634	*Acinetobacter baumannii*	Acinetobacter baumannii isolate 2024DU00030 SAMN44842723-rid24048333.denovo.10	100%	100%	ABWLHP010000010.1
635	*Acinetobacter baumannii*	Acinetobacter baumannii isolate 2024DU00025 SAMN44780859-rid23985503.denovo.12	100%	100%	ABWKKP010000012.1
636	*Acinetobacter baumannii*	Acinetobacter baumannii isolate 2024DU00024 SAMN44780854-rid23985513.denovo.39	100%	100%	ABWKKN010000035.1
637	*Acinetobacter baumannii*	Acinetobacter baumannii isolate 2024DU00022 SAMN44780858-rid23985533.denovo.06	100%	100%	ABWKKL010000006.1
638	*Acinetobacter baumannii*	Acinetobacter baumannii isolate 2024DU00021 SAMN44780857-rid23985543.denovo.13	100%	100%	ABWKKJ010000013.1
639	*Acinetobacter baumannii*	Acinetobacter baumannii isolate 2024DU00019 SAMN44780849-rid23985563.denovo.11	100%	100%	ABWKKI010000011.1
640	*Acinetobacter baumannii*	Acinetobacter baumannii isolate 2024DU00018 SAMN44780847-rid23985573.denovo.11	100%	100%	ABWKKG010000011.1
641	*Acinetobacter baumannii*	Acinetobacter baumannii isolate 2024DU00012 SAMN44780850-rid23985633.denovo.10	100%	100%	ABWKKB010000010.1
642	*Acinetobacter baumannii*	Acinetobacter baumannii isolate 2024DU00011 SAMN44771306-rid23982383.denovo.11	100%	100%	ABWKAR010000011.1
643	*Acinetobacter baumannii*	Acinetobacter baumannii isolate 2024DU00010 SAMN44771305-rid23981903.denovo.09	100%	100%	ABWKAN010000009.1
644	*Acinetobacter baumannii*	Acinetobacter baumannii isolate 2024DU00007 SAMN44771308-rid23980713.denovo.11	100%	100%	ABWKDI010000011.1
645	*Acinetobacter baumannii*	Acinetobacter baumannii isolate 2024DU00002 SAMN44409154-rid23672743.denovo.10	100%	100%	ABVZRO010000010.1
646	*Acinetobacter baumannii*	Acinetobacter baumannii isolate 2024DU00001 SAMN44409153-rid23672753.denovo.12	100%	100%	ABVZTY010000012.1
647	*Acinetobacter baumannii*	Acinetobacter baumannii isolate 2024CB-00274 SAMN41072653-rid21493043.denovo.51	100%	100%	ABRWWL010000045.1
648	*Acinetobacter baumannii*	Acinetobacter baumannii isolate 2024BM-00212 SAMN45230641-rid24402083.denovo.12	100%	100%	ABWQPE010000012.1
649	*Acinetobacter baumannii*	Acinetobacter baumannii isolate 2024BM-00206 SAMN45033488-rid24176493.denovo.11	100%	100%	ABWMJK010000011.1
650	*Acinetobacter baumannii*	Acinetobacter baumannii isolate 2024BM-00205 SAMN45033487-rid24176503.denovo.12	100%	100%	ABWMJL010000012.1
651	*Acinetobacter baumannii*	Acinetobacter baumannii isolate 2024BM-00204 SAMN45033486-rid24176513.denovo.12	100%	100%	ABWMJJ010000012.1
652	*Acinetobacter baumannii*	Acinetobacter baumannii isolate 2024BM-00199 SAMN44769615-rid23978353.denovo.13	100%	100%	ABWJTW010000013.1
653	*Acinetobacter baumannii*	Acinetobacter baumannii isolate 2024BM-00191 SAMN44696121-rid23951563.denovo.12	100%	100%	ABWITD010000012.1
654	*Acinetobacter baumannii*	Acinetobacter baumannii isolate 2024BM-00190 SAMN44696120-rid23951573.denovo.13	100%	100%	ABWITA010000013.1
655	*Acinetobacter baumannii*	Acinetobacter baumannii isolate 2024BM-00179 SAMN44406147-rid23668363.denovo.11	100%	100%	ABVZUH010000011.1
656	*Acinetobacter baumannii*	Acinetobacter baumannii isolate 2024BM-00171 SAMN44294263-rid23630023.denovo.11	100%	100%	ABVXEY010000011.1
657	*Acinetobacter baumannii*	Acinetobacter baumannii isolate 2024BM-00162 SAMN44473005-rid23764843.denovo.11	100%	100%	ABWAXK010000011.1
658	*Acinetobacter baumannii*	Acinetobacter baumannii isolate 2024BM-00156 SAMN43942835-rid23462013.denovo.11	100%	100%	ABVDNA010000011.1
659	*Acinetobacter baumannii*	Acinetobacter baumannii isolate 2024BM-00154 SAMN43942833-rid23462033.denovo.11	100%	100%	ABVDMZ010000011.1
660	*Acinetobacter baumannii*	Acinetobacter baumannii isolate 2024BM-00150 SAMN43785709-rid23380173.denovo.11	100%	100%	ABUXIJ010000011.1
661	*Acinetobacter baumannii*	Acinetobacter baumannii isolate 2024BM-00149 SAMN43785708-rid23380473.denovo.11	100%	100%	ABUXII010000011.1
662	*Acinetobacter baumannii*	Acinetobacter baumannii isolate 2024BM-00147 SAMN43795456-rid23397473.denovo.12	100%	100%	ABUXCJ010000012.1
663	*Acinetobacter baumannii*	Acinetobacter baumannii isolate 2024BM-00136 SAMN43366224-rid23011123.denovo.12	100%	100%	ABUHPQ010000012.1
664	*Acinetobacter baumannii*	Acinetobacter baumannii isolate 2024BM-00117 SAMN42890655-rid22481933.denovo.11	100%	100%	ABTSGU010000011.1
665	*Acinetobacter baumannii*	Acinetobacter baumannii isolate 2024BM-00105 SAMN42493429-rid22317073.denovo.45	100%	100%	ABTMXA010000040.1
666	*Acinetobacter baumannii*	Acinetobacter baumannii isolate 2024BM-00070 SAMN41506038-rid21702613.denovo.10	100%	100%	ABSKKK010000010.1
667	*Acinetobacter baumannii*	Acinetobacter baumannii isolate 2024BM-00056 SAMN41079607-rid21496283.denovo.12	100%	100%	ABRWUO010000012.1
668	*Acinetobacter baumannii*	Acinetobacter baumannii isolate 2024BM-00053 SAMN41019501-rid21472713.denovo.13	100%	100%	ABRVCM010000013.1
669	*Acinetobacter baumannii*	Acinetobacter baumannii isolate 2024BM-00046 SAMN40969012-rid21439233.denovo.12	100%	100%	ABRSCD010000012.1
670	*Acinetobacter baumannii*	Acinetobacter baumannii isolate 2024BM-00045 SAMN40928866-rid21428093.denovo.13	100%	100%	ABRREA010000013.1
671	*Acinetobacter baumannii*	Acinetobacter baumannii isolate 2024BM-00044 SAMN40928865-rid21428103.denovo.13	100%	100%	ABRREE010000013.1
672	*Acinetobacter baumannii*	Acinetobacter baumannii isolate 2024BM-00034 SAMN40575151-rid21311713.denovo.11	100%	100%	ABQWWH010000011.1
673	*Acinetobacter baumannii*	Acinetobacter baumannii isolate 2024BM-00008 SAMN39530267-rid20775773.denovo.13	100%	100%	ABPYHL010000013.1
674	*Acinetobacter baumannii*	Acinetobacter baumannii isolate 2024BM-00001 SAMN39457990-rid20737453.denovo.14	100%	100%	ABPXDT010000014.1
675	*Acinetobacter baumannii*	Acinetobacter baumannii isolate 2023DK-00305 SAMN37997772-rid20863923.denovo.10	100%	100%	ABOKET020000010.1
676	*Acinetobacter baumannii*	Acinetobacter baumannii isolate 2023BM-00187 SAMN38909868-rid20754403.denovo.11	100%	100%	ABPVXD010000011.1
677	*Acinetobacter baumannii*	Acinetobacter baumannii isolate 2023BM-00186 SAMN38842850-rid20583323.denovo.12	100%	100%	ABPLPW010000012.1
678	*Acinetobacter baumannii*	Acinetobacter baumannii isolate 2023BM-00135 SAMN37684926-rid20929963.denovo.11	100%	100%	ABNLPP020000011.1
679	*Acinetobacter baumannii*	Acinetobacter baumannii isolate 2023BM-00134 SAMN37684925-rid20940863.denovo.10	100%	100%	ABNLTX020000010.1
680	*Acinetobacter baumannii*	Acinetobacter baumannii isolate 2023BM-00121 SAMN37297685-rid20941843.denovo.12	100%	100%	ABMCHP020000012.1
681	*Acinetobacter baumannii*	Acinetobacter baumannii isolate 2023BM-00116 SAMN37297680-rid20907893.denovo.11	100%	100%	ABMCHK020000011.1
682	*Acinetobacter baumannii*	Acinetobacter baumannii isolate 2023BM-00076 SAMN35995875-rid20938233.denovo.11	100%	100%	ABLZHQ020000011.1
683	*Acinetobacter baumannii*	Acinetobacter baumannii isolate 2023BM-00071 SAMN35995871-rid20894533.denovo.11	100%	100%	ABLZHP020000011.1
684	*Acinetobacter baumannii*	Acinetobacter baumannii isolate 2023BM-00049 SAMN34352570-rid20930443.denovo.12	100%	100%	ABLZXE020000012.1
685	*Acinetobacter baumannii*	Acinetobacter baumannii isolate 2023BM-00027 SAMN33569646-rid20866763.denovo.11	100%	100%	ABKWFX030000011.1
686	*Acinetobacter baumannii*	Acinetobacter baumannii isolate 2022EP-00180 SAMN32308517-rid20872503.denovo.09	100%	100%	ABKGAS030000009.1
687	*Acinetobacter baumannii*	Acinetobacter baumannii isolate 2022EP-00091 SAMN30651436-rid20892983.denovo.12	100%	100%	ABKGEB030000012.1
688	*Acinetobacter baumannii*	Acinetobacter baumannii isolate 2022EP-00086 SAMN29886483-rid20850693.denovo.10	100%	100%	ABKGEL030000010.1
689	*Acinetobacter baumannii*	Acinetobacter baumannii isolate 2022BM-00184 SAMN34029926-rid20635073.denovo.11	100%	100%	ABLIGQ030000011.1
690	*Acinetobacter baumannii*	Acinetobacter baumannii isolate 2022BM-00179 SAMN34030559-rid20905443.denovo.11	100%	100%	ABLMRS030000011.1
691	*Acinetobacter baumannii*	Acinetobacter baumannii isolate 2021EP-00087 SAMN25330379-rid20852243.denovo.035	100%	100%	ABKGKC030000034.1
692	*Acinetobacter baumannii*	Acinetobacter baumannii isolate 2005569 SAMN30321860-rid24927863.denovo.40	100%	100%	ABXEDG010000037.1
693	*Acinetobacter baumannii*	Acinetobacter baumannii isolate 2001537 SAMN30321842-rid24928333.denovo.40	100%	100%	ABXEEF010000037.1
694	*Acinetobacter baumannii*	Acinetobacter baumannii isolate 2001533 SAMN30321840-rid24928353.denovo.39	100%	100%	ABXEED010000035.1
695	*Acinetobacter baumannii*	Acinetobacter baumannii isolate 2001532 SAMN30321884-rid24927743.denovo.42	100%	100%	ABXEDQ010000036.1
696	*Acinetobacter baumannii*	Acinetobacter baumannii isolate 04A15CRGN029 SAMN07258621-rid10848253.denovo.42	100%	100%	DADADT010000039.1
697	*Acinetobacter baumannii*	Acinetobacter baumannii strain J9 plasmid pJ9-3, complete sequence	100%	100%	CP041590.1
698	*Acinetobacter baumannii*	Acinetobacter baumannii strain 728D plasmid pAba728Dc, complete sequence	100%	100%	CP191619.1
699	*Acinetobacter baumannii*	Acinetobacter baumannii strain AB177-VUB chromosome, complete genome	100%	100%	CP091361.1
700	*Acinetobacter baumannii*	Acinetobacter baumannii strain WCHAB005078 plasmid pOXA58_005078, complete sequence	100%	100%	CP027245.2

701	*Acinetobacter baumannii*	Acinetobacter baumannii strain DETAB-E227 plasmid pDETAB5, complete sequence	100%	100%	CP072528.1
702	*Acinetobacter baumannii*	Acinetobacter baumannii strain AB186-VUB chromosome, complete genome	100%	100%	CP091356.1
703	*Acinetobacter baumannii*	Acinetobacter baumannii strain 2016GDAB1 plasmid p5637, complete sequence	100%	100%	CP065052.1
704	*Acinetobacter bereziniae*	Acinetobacter bereziniae strain MM20190808-2-1 NODE_9_length_5381_cov_1077.38	100%	100%	JAOXHQ010000009.1
705	*Acinetobacter bereziniae*	Acinetobacter bereziniae LMG 1003 = CIP 70.12 acLsv-supercont1.1.C4	100%	100%	APQG01000004.1
705	*Acinetobacter bereziniae*	Acinetobacter bereziniae LMG 1003 = CIP 70.12 acLsv-supercont1.1.C3	100%	100%	APQG01000003.1
706	*Acinetobacter faecalis*	Acinetobacter portensis strain JNE5 plasmid pJNE5-X3_NDM-1, complete sequence	100%	100%	CP096121.1
707	*Acinetobacter indicus*	Acinetobacter sp. YH12230 NODE_28_length_5909_cov_42.233310	100%	100%	VPDI01000028.1
708	*Acinetobacter indicus*	Acinetobacter sp. YH12217 NODE_26_length_5909_cov_91.395884	100%	100%	VPDA01000026.1
709	*Acinetobacter indicus*	Acinetobacter sp. YH12216 NODE_26_length_5909_cov_90.788309	100%	100%	VPCZ01000026.1
710	*Acinetobacter indicus*	Acinetobacter sp. YH12214 NODE_28_length_5909_cov_85.170356	100%	100%	VPCY01000028.1
711	*Acinetobacter indicus*	Acinetobacter sp. YH12212 NODE_26_length_5909_cov_86.723106	100%	100%	VPCX01000026.1
712	*Acinetobacter indicus*	Acinetobacter indicus strain DFZJA80-1 NODE_40_length_11756_cov_305.232391	100%	100%	RZUA01000037.1
713	*Acinetobacter indicus*	Acinetobacter indicus strain FS42-2 chromosome, complete genome	100%	100%	CP046595.1
714	*Acinetobacter indicus*	Acinetobacter indicus strain C15_T plasmid pC15-1, complete sequence	100%	100%	CP048655.1
715	*Acinetobacter indicus*	Acinetobacter indicus strain CMG3-2 plasmid pCMG3-2-3, complete sequence	100%	100%	CP044448.1
716	*Acinetobacter indicus*	Acinetobacter indicus strain B18 plasmid pB18-2, complete sequence	100%	100%	CP044457.1
717	*Acinetobacter indicus*	Acinetobacter indicus strain GXNN15X4 plasmid pGXNN15, complete sequence	100%	100%	CP071318.1
718	*Acinetobacter johnsonii*	Acinetobacter johnsonii strain 18QD2AZ57W 139_37_length_14041_cov_432.103	100%	100%	WURO01000037.1
719	*Acinetobacter johnsonii*	Acinetobacter johnsonii strain ASP1934 NODE_49_length_10421_cov_143.632927	100%	100%	JAZHCN010000049.1
720	*Acinetobacter johnsonii*	Acinetobacter johnsonii strain FAHZZU3110hy plasmid pFAHZZU3110hy-2, complete sequence	100%	100%	CP140716.1
721	*Acinetobacter johnsonii*	Acinetobacter johnsonii strain FAHZZU3567hy plasmid pFAHZZU3567hy-2, complete sequence	100%	100%	CP140709.1
722	*Acinetobacter lwoffii*	Acinetobacter lwoffii strain 72 contig_26	100%	100%	JBMPXW010000009.1
723	*Acinetobacter nosocomialis*	Acinetobacter nosocomialis strain AN1 2	100%	100%	JACLCR010000002.1
724	*Acinetobacter nosocomialis*	Acinetobacter nosocomialis strain E9 contig_22_len_29889	100%	100%	JBKJBV010000022.1
725	*Acinetobacter nosocomialis*	Acinetobacter nosocomialis strain E8 contig_21_len_29889	100%	100%	JBKJBW010000021.1
726	*Acinetobacter nosocomialis*	Acinetobacter nosocomialis strain E7 contig_22_len_29889	100%	100%	JBKJBX010000022.1
727	*Acinetobacter nosocomialis*	Acinetobacter nosocomialis strain E6 contig_21_len_29889	100%	100%	JBKJBY010000021.1
728	*Acinetobacter nosocomialis*	Acinetobacter nosocomialis strain E4 contig_23_len_29889	100%	100%	JBKJBZ010000023.1
729	*Acinetobacter nosocomialis*	Acinetobacter nosocomialis strain E2 contig_22_len_29889	100%	100%	JBKJCA010000022.1
730	*Acinetobacter nosocomialis*	Acinetobacter nosocomialis strain E16 contig_21_len_29889	100%	100%	JBKJBO010000021.1
731	*Acinetobacter nosocomialis*	Acinetobacter nosocomialis strain E15 contig_22_len_29889	100%	100%	JBKJBP010000022.1
732	*Acinetobacter nosocomialis*	Acinetobacter nosocomialis strain E14 contig_22_len_29889	100%	100%	JBKJBQ010000022.1
733	*Acinetobacter nosocomialis*	Acinetobacter nosocomialis strain E13 contig_21_len_29889	100%	100%	JBKJBR010000021.1
734	*Acinetobacter nosocomialis*	Acinetobacter nosocomialis strain E12 contig_22_len_29889	100%	100%	JBKJBS010000022.1
735	*Acinetobacter nosocomialis*	Acinetobacter nosocomialis strain E1 contig_21_len_29889	100%	100%	JBKJCB010000021.1
736	*Acinetobacter nosocomialis*	Acinetobacter nosocomialis strain Ab122 genome assembly, contig: NODE_23_length_10827_cov_22.342569	100%	100%	UCQC02000061.1
737	*Acinetobacter nosocomialis*	Acinetobacter nosocomialis strain 6B contig_22_len_29889	100%	100%	JBKJCC010000022.1
738	*Acinetobacter nosocomialis*	Acinetobacter nosocomialis strain 5B contig_22_len_29889	100%	100%	JBKJCD010000022.1
739	*Acinetobacter nosocomialis*	Acinetobacter nosocomialis strain 4B contig_22_len_29889	100%	100%	JBKJCE010000022.1
740	*Acinetobacter nosocomialis*	Acinetobacter nosocomialis strain 3B contig_23_len_29889	100%	100%	JBKJCF010000023.1
741	*Acinetobacter nosocomialis*	Acinetobacter nosocomialis strain 2B contig_24_len_24994	100%	100%	JBKJCG010000024.1
742	*Acinetobacter nosocomialis*	Acinetobacter nosocomialis strain 27B contig_24_len_29889	100%	100%	JBKJBN010000024.1
743	*Acinetobacter nosocomialis*	Acinetobacter nosocomialis strain 1B contig_22_len_29889	100%	100%	JBKJCH010000022.1
744	*Acinetobacter nosocomialis*	Acinetobacter nosocomialis strain 11J360 plasmid p11J360-1, complete sequence	100%	100%	CP095868.1
745	*Acinetobacter pittii*	Acinetobacter pittii strain T822-1 NODE_16_length_35625_cov_228.429194	100%	100%	JAHNAU010000016.1
746	*Acinetobacter pittii*	Acinetobacter pittii strain T820 NODE_17_length_35625_cov_225.084477	100%	100%	JAHNAT010000017.1
747	*Acinetobacter pittii*	Acinetobacter pittii strain WZ-38 WZ-38_29_47785_cov_34.373620	100%	100%	JBDQPP010000043.1
748	*Acinetobacter pittii*	Acinetobacter pittii strain R820 NODE_19_length_35625_cov_227.271548	100%	100%	JAHMWB010000019.1
749	*Acinetobacter pittii*	Acinetobacter pittii strain mNGS-11_59 tig00000005	100%	100%	JAQAJH010000005.1
750	*Acinetobacter pittii*	Acinetobacter pittii strain mNGS-11_33 tig00000002	100%	100%	JAQAJF010000002.1
751	*Acinetobacter pittii*	Acinetobacter pittii strain 1602.027 Scaffold18	100%	100%	JASIWM010000018.1
752	*Acinetobacter pseudolwoffii*	Acinetobacter sp. YH12200 NODE_46_length_6322_cov_86.639548	100%	100%	VPCO01000048.1
753	*Acinetobacter pseudolwoffii*	Acinetobacter sp. YH12156 NODE_21_length_8060_cov_528.135636	100%	100%	VPCL01000024.1
754	*Acinetobacter pseudolwoffii*	Acinetobacter sp. YH12151 NODE_55_length_11972_cov_48.121570	100%	100%	VPCG01000057.1
755	*Acinetobacter pseudolwoffii*	Acinetobacter sp. YH01008 NODE_57_length_6322_cov_86.147215	100%	100%	VOYD01000059.1
756	*Acinetobacter pseudolwoffii*	Acinetobacter pseudolwoffii strain XMC5X702 plasmid pXMC5X702-tetX-145k, complete sequence	100%	100%	CP084302.1
757	*Acinetobacter schindleri*	Acinetobacter schindleri strain HZE23-1 plasmid pHZE23-1-8, complete sequence	100%	100%	CP044471.1
757	*Acinetobacter schindleri*	Acinetobacter schindleri strain HZE23-1 plasmid pHZE23-1-4, complete sequence	100%	100%	CP044467.1
758	*Acinetobacter schindleri*	Acinetobacter schindleri strain HZE33-1 plasmid pHZE33-1-4, complete sequence	100%	100%	CP044478.1
759	*Acinetobacter towneri*	Acinetobacter towneri strain DF29-5 NODE_29_length_2410_cov_174.272182	100%	100%	JACANF010000029.1
760	*Acinetobacter towneri*	Acinetobacter sp. YH16053 NODE_49_length_5140_cov_3285.330142	100%	100%	VPEP01000050.1
761	*Acinetobacter towneri*	Acinetobacter sp. YH12058 NODE_27_length_24678_cov_82.184025	100%	100%	VOZL01000027.1
762	*Acinetobacter towneri*	Acinetobacter sp. YH01013 NODE_17_length_10614_cov_250.494422	100%	100%	VOYI01000017.1
763	*Acinetobacter towneri*	Acinetobacter sp. YH01002 NODE_10_length_41164_cov_54.339084	100%	100%	VOXX01000010.1
764	*Acinetobacter ursingii*	Acinetobacter ursingii TUM15523 DNA, sequence035	100%	100%	BKWZ01000035.1
765	*Acinetobacter ursingii*	Acinetobacter ursingii TUM15519 DNA, sequence037	100%	100%	BKWV01000037.1
766	*Acinetobacter ursingii*	Acinetobacter ursingii TUM15518 DNA, sequence046	100%	100%	BKWU01000046.1
767	*Acinetobacter ursingii*	Acinetobacter ursingii TUM15098 DNA, sequence049	100%	100%	BKHC01000049.1
768	*Acinetobacter variabilis*	Acinetobacter variabilis strain BDT2044 plasmid pBDT2044-2, complete sequence	100%	100%	CP094248.1
769	*Acinetobacter variabilis*	Acinetobacter variabilis strain XM9F202-2 plasmid pXM9F202-2-186k, complete sequence	100%	100%	CP060812.1
770	*Acinetobacter yuyunsongii*	Acinetobacter sp. XH1639 plasmid pXH1639-OXA, complete sequence	100%	100%	CP157437.1
771	*Acinetobacter yuyunsongii*	Acinetobacter sp. WCHA45 plasmid pNDM1_010045, complete sequence	100%	100%	CP028560.1
772	*Candidatus* Acinetobacter avistercoris	Acinetobacter sp. KS-LM10 plasmid unnamed1	100%	100%	CP145907.1
773	Taxon 58	Acinetobacter sp. YH16032 NODE_117_length_1963_cov_169.208061	100%	100%	VPEE01000120.1
774	Taxon 58	Acinetobacter sp. YH12239 NODE_33_length_8862_cov_325.431139	100%	100%	VPDP01000035.1
775	Taxon 76	Acinetobacter sp. YH12210 NODE_83_length_10366_cov_145.578865	100%	100%	VPCV01000087.1
776	Taxon 76	Acinetobacter sp. YH12209 NODE_88_length_10366_cov_141.744506	100%	100%	VPCU01000091.1
777	Taxon 76	Acinetobacter sp. YH12207 NODE_88_length_10366_cov_134.407462	100%	100%	VPCS01000091.1
778	Taxon 76	Acinetobacter sp. YH12206 NODE_85_length_10366_cov_159.093466	100%	100%	VPCR01000088.1
779	Taxon 76	Acinetobacter sp. 251-1 NODE_35_length_9915_cov_490.532019	100%	100%	JACANS010000035.1
780	Taxon 83	Acinetobacter indicus strain DF100-5 NODE_61_length_8812_cov_1169.242129	100%	100%	JACANX010000061.1
781	Taxon 96	Acinetobacter sp. YH12047 NODE_44_length_6322_cov_152.168523	100%	100%	VOZG01000046.1
